# Visual and modular detection of pathogen nucleic acids with enzyme–DNA molecular complexes

**DOI:** 10.1038/s41467-018-05733-0

**Published:** 2018-08-13

**Authors:** Nicholas R. Y. Ho, Geok Soon Lim, Noah R. Sundah, Diana Lim, Tze Ping Loh, Huilin Shao

**Affiliations:** 1 Biomedical Institute for Global Health Research and Technology, National University of Singapore, Singapore, 117599 Singapore; 2 Institute of Molecular and Cell Biology, Agency for Science, Technology and Research, Singapore, 138673 Singapore; 3 Department of Biomedical Engineering, Faculty of Engineering, National University of Singapore, Singapore, 117583 Singapore; 4 Department of Pathology, National University Hospital, Singapore, 119074 Singapore; 5 Department of Laboratory Medicine, National University Hospital, Singapore, 117599 Singapore; 6 Department of Surgery, Yong Loo Lin School of Medicine, National University of Singapore, Singapore, 119228 Singapore

## Abstract

Rapid, visual detection of pathogen nucleic acids has broad applications in infection management. Here we present a modular detection platform, termed enzyme-assisted nanocomplexes for visual identification of nucleic acids (enVision). The system consists of an integrated circuit of enzyme–DNA nanostructures, which function as independent recognition and signaling elements, for direct and versatile detection of pathogen nucleic acids from infected cells. The built-in enzymatic cascades produce a rapid color readout for the naked eye; the assay is thus fast (<2 h), sensitive (<10 amol), and readily quantified with smartphones. When implemented on a configurable microfluidic platform, the technology demonstrates superior programmability to perform versatile computations, for detecting diverse pathogen targets and their virus–host genome integration loci. We further design the enVision platform for molecular-typing of infections in patient endocervical samples. The technology not only improves the clinical inter-subtype differentiation, but also expands the intra-subtype coverage to identify previously undetectable infections.

## Abstract (web summary)

Rapid, visual detection of pathogens is important for point-of-care diagnostics. Here the authors present enVision, which uses enzyme-DNA complexes to detect pathogen nucleic acids and provide a rapid, smartphone compatible readout.

## Introduction

Detection of pathogen nucleic acids has broad applications in infection diagnostics and management. As an alternative to conventional pathogen culture, which entails long processing time (i.e., several days) and requires species-specific protocols (e.g., bacteria vs. viruses), nucleic acid technologies have been increasingly adopted in clinical laboratories to provide unprecedented molecular information about infections (and beyond)^1–3^. For example, nucleic acid-based human papillomavirus (HPV) testing is essential to contemporary cervical cancer testing. HPV, the most common sexually transmitted infection, is the primary cause of cervical cancer^4^. There are >100 subtypes of HPV, of which 15 are considered of high malignancy risk^5^. HPV infection is a global epidemic; while mostly benign, some of these infections can progress to cause deadly cervical cancer. This complex etiology, carcinogenesis and disease progression are primarily linked to two factors: (1) infection from specific HPV molecular subtypes, and (2) the persistence of infection^6,7^. Point-of-care testing that can distinguish the infection subtypes and be performed at the patient level (e.g., community clinics and doctor’s offices)^8–11^ could thus bring tremendous opportunities for patient stratification and accessible monitoring, and is associated with better health outcomes^12^.

Current detection of pathogen nucleic acids, however, is almost exclusively performed in large centralized clinical laboratories. This limited reach arises from the high complexity and cost associated with conventional technologies. In the case of HPV detection, commercial assays leverage primarily on polymerase chain reaction (PCR, e.g., Cobas HPV) to amplify and detect specific DNA targets^13,14^. Such systems not only necessitate bulky and specialized equipment, for PCR thermal cycling and fluorescence measurements, but also require trained personnel to operate. Advanced isothermal amplification assays have been developed to relieve the instrument needs for temperature cycling; nevertheless, these assays have their own limitations. For example, loop-mediated isothermal amplification (LAMP) has stringent sequence requirements and cannot be easily generalized^15^. Importantly, as with other nucleic acid amplification approaches, LAMP is prone to false-positives (e.g., from primer-dimer formation). Alternatively, sequence-specific signaling probes (e.g., fluorescent Taqman reporter) could be used to improve the detection accuracy; however, these probes are expensive and complex to implement^16^. As each piece of DNA target requires a dedicated, sequence-specific probe for coupled signaling during target amplification, the approach becomes increasingly costly and challenging to multiplex or perform complex computations^17^.

To address these challenges, we developed a molecular platform to enable visual and modular detection of diverse pathogen nucleic acids. Instead of relying on target nucleic acid amplification, as in the above-mentioned approaches, the technology exponentially enhances visual signal from direct and independent target hybridization. Termed enzyme-assisted nanocomplexes for visual identification of nucleic acids (enVision), the technology consists of an integrated circuit of two independent enzyme–DNA nanostructures—an easily adjustable recognition element and a sensitive universal signaling element—to decouple target recognition and visual signal amplification. We chose DNA nanostructures as the functional elements, as they can be designed to harbor stable three-dimensional conformations to facilitate diverse enzymatic activities, and have minimal cross-talk, even when packed closely, to enable independent operations^18–20^.

## Results

### enVision platform

**Fig. 1:**
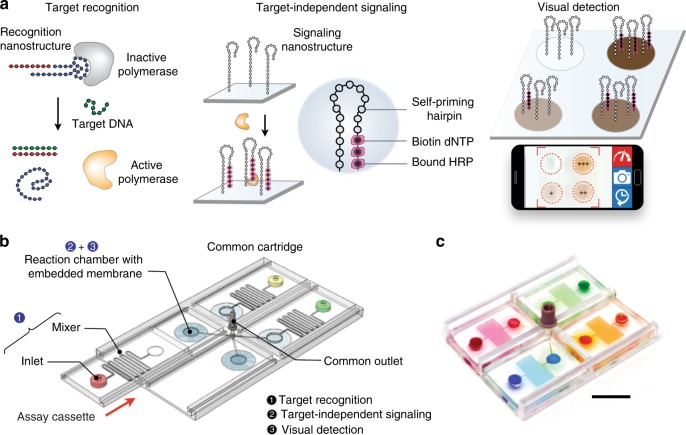
Visual and modular detection of pathogen nucleic acids. **a** The enVision system consists of a series of enzyme–DNA nanostructures to enable target recognition, target-independent signaling, and visual detection. The nanostructures are designed to decouple recognition from signaling. The recognition nanostructure is a hybrid complex, composed of an inactivating aptamer and a Taq DNA polymerase. In the presence of complementary target DNA, the complex dissociates to activate the polymerase activity. The active polymerase proceeds to elongate a universal, self-priming signaling nanostructure, in a target-independent manner. Modified deoxynucleotides (dNTPs) are incorporated to immobilize horseradish peroxidase (HRP) onto the signaling nanostructures. Upon the addition of optical substrate, visual signals can be enzymatically enhanced, detected by the naked eye and quantified with a smartphone camera. Photograph (inset) shows an example of the actual visual readouts in the presence of none (−) and varying (+) amounts of target DNA on a smartphone application. **b** Schematic of the enVision microfluidic system. The platform is designed to complement the modular enVision workflow. Independent assay cassettes, preloaded with specific recognition nanostructures at the inlets, can be mounted on-demand onto a common signaling cartridge. The common cartridge houses the universal signaling nanostructures, which are immobilized on embedded membranes, for target-independent signaling and visual detection. Direction of cassette sliding is indicated by a red arrow. **c** Photograph of the microfluidic enVision prototype, developed for versatile assay integration and parallel processing. Scale bar indicates 1 cm

The enVision platform consists of a series of enzyme-assisted DNA nanostructures to achieve three functional steps: DNA target recognition, target-independent signal enhancement, and visual detection (Fig. 1a). With orthogonal sequence design of the nanostructures, the target recognition is decoupled from the signal enhancement. In the recognition step, the recognition element is a unique hybrid nanostructure. It consists of a modified DNA aptamer^21,22^ bound to a Taq DNA polymerase. In the absence of target DNA, the aptamer binds strongly with the polymerase to inhibit polymerase activity. In the presence of complementary target DNA, upon target hybridization, the hybrid dissociates to activate polymerase activity. In the signaling step, the active polymerase elongates a universal, self-priming nanostructure, in a target-independent manner. Through the incorporation of modified oligonucleotides into the signaling structures, visual signals can be enzymatically produced for detection by the naked eye and are readily quantified through smartphones. The insert (Fig. 1a, right) shows an example of the actual assay output as imaged by smartphones.

To complement the detection programmability and modularity, we implemented the enVision assays in a configurable microfluidic platform (Fig. 1b). Specifically, we integrated two components: (i) independent assay cassettes, and (ii) a common signaling cartridge. Each assay cassette was preloaded with specific DNA recognition nanostructures, and could be plugged in on-demand to enable versatile assay integration. The common holder cartridge housed the universal signaling elements, which were immobilized on polycarbonate membranes. Fluid flow and sample mixing were performed through a parallel circuit of microchannels (Supplementary Fig. 1) and actuated by negative pressure at a common outlet on the cartridge to streamline the assay procedure (Supplementary Fig. 2). Figure 1c shows a prototype device developed for clinical detection of pathogen DNA targets. This system compartmentalization improves the assay modularity (i.e., on-demand integration) and simplifies the implementation (i.e., uniform surface immobilization and single flow actuation)^23^ for clinical applications.

### Optimized assay for visual quantification of nucleic acids

**Fig. 2:**
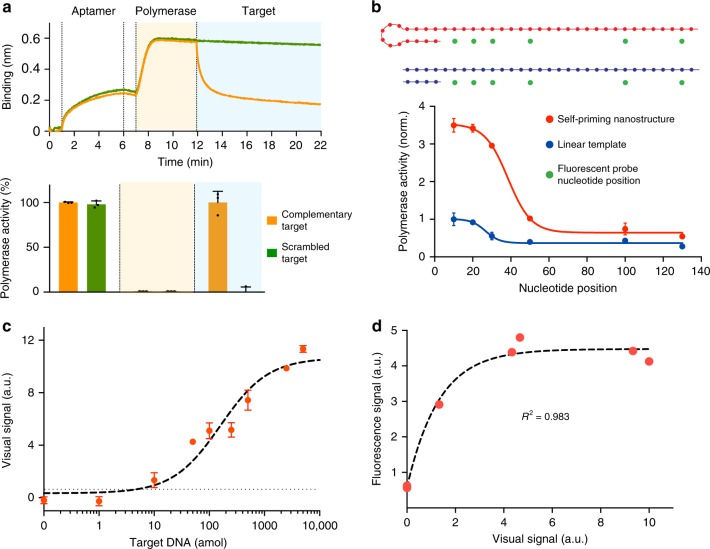
Nucleic acid quantification with enVision. **a** Recognition nanostructure assembly and activity. The recognition nanostructure was assembled and incubated with complementary or scrambled target DNA sequences, to determine the resultant polymerase association and activity. (Top) Real-time sensorgram of molecular binding. We performed a series of operations, namely aptamer immobilization, addition of polymerase, and incubation with target DNA sequences. Molecular binding was monitored in situ through bio-layer interferometry to determine polymerase association. (Bottom) The corresponding polymerase activity was determined at the end of each operation via a parallel experiment using a Taqman assay (fluorescence measurement of 5′ exonuclease degradation of Taqman probes). Note the complete recovery of polymerase activity upon incubating with complementary target DNA. **b** Signaling nanostructure activity. In a comparative experiment, the self-priming signaling nanostructure and its similarly-sized linear template were treated with equal concentration of active DNA polymerase. The polymerase activity was determined at different nucleotide positions away from the starting primed sites, through 5′ exonuclease degradation of differentially placed Taqman probes (positions indicated as green dots). **c** Detection sensitivity of the enVision system. The detection limit (dotted line) was determined by titrating a known amount of target DNA and measuring their associated visual signals. All visual signals were acquired through a smartphone. **d** Correlation between the enVision and fluorescence measurements on varying quantities of target DNA. The visual signal matched well with the fluorescence signal (*R*^2^ = 0.9828) and demonstrated a wider dynamic range. All measurements were performed in triplicate, and the data are displayed as mean ± s.d. a.u. arbitrary unit

We first evaluated the performance of the DNA nanostructures as functional recognition and signaling elements, respectively. For the recognition nanostructure, the polymerase associated with the modified aptamer to form a hybrid complex (Fig. 2a, top). This optimized complex demonstrated negligible polymerase activity (Supplementary Fig. 3a). In the presence of complementary target DNA, the hybrid structure dissociated and the polymerase activity recovered fully (Fig. 2a, bottom). We found that this recognition and activation showed a high sequence specificity, as only complementary targets resulted in strong polymerase activity, while scrambled oligonucleotide sequences produced negligible activity (Supplementary Fig. 3b).

We further designed the universal signaling element as a self-priming nanostructure. As compared to its similarly-sized linear counterpart, the structure demonstrated enhanced polymerase occupancy and activity (Fig. 2b), possibly due to its superior priming efficiency at room temperature (Supplementary Fig. 4). All sequences used to optimize the assembly and characterization of the nanostructures can be found in Supplementary Table 1.

We subsequently integrated these independent elements to develop the enVision workflow. To enable visual detection, we used the activated polymerase to elongate the signaling nanostructure and incorporate horseradish peroxidase (HRP) through biotinylated nucleotides (biotin-16-AA-dCTP). We further optimized both the polymerase and HRP reactions (Supplementary Fig. 5a). In the presence of optical substrate, the HRP activity developed brown precipitates rapidly. In a titration analysis, we observed that the color intensity could be image-quantified and correlated to increasing amounts of target DNA (Supplementary Fig. 5b). When imaged with a smartphone camera, the visual assay demonstrated a limit of detection (LOD) of 0.260 fmol of target DNA (Supplementary Fig. 5c) and the assay could be completed in as little as 30 min at room temperature. The LOD could be further improved to 7.205 amol of target DNA (Fig. 2c) through integration with efficient asymmetric amplification (Supplementary Fig. 6). Isothermal asymmetric amplification through nucleic acid sequence based amplification (NASBA)^9^ could also be incorporated to improve practical applications (Supplementary Fig. 7). All primers and targets used for amplification can be found in Supplementary Table 1. Detection sensitivities were determined with point-of-care smartphone readouts; all processes (including asymmetric amplification and NASBA) were accomplished in <2 h.

To correlate the visual signal with standard fluorescence signal, we performed a similar experiment with FAM-modified nucleotides (Supplementary Fig. 5d). The enVision visual assay correlated well to a logistic function that accounts for fluorescence signal saturation (*R*^2^ = 0.983) (Fig. 2d). When compared to the fluorescence measurements, which saturated at high concentrations of DNA targets, the enVision platform showed a wider dynamic range to produce distinguishable and quantifiable visual signals at these target concentrations. We attribute this expansion in the visual dynamic range to the incorporation of dual enzymatic signaling (i.e., DNA polymerase and HRP cascades) in the enVision technology.

### Programmability of enVision

**Fig. 3:**
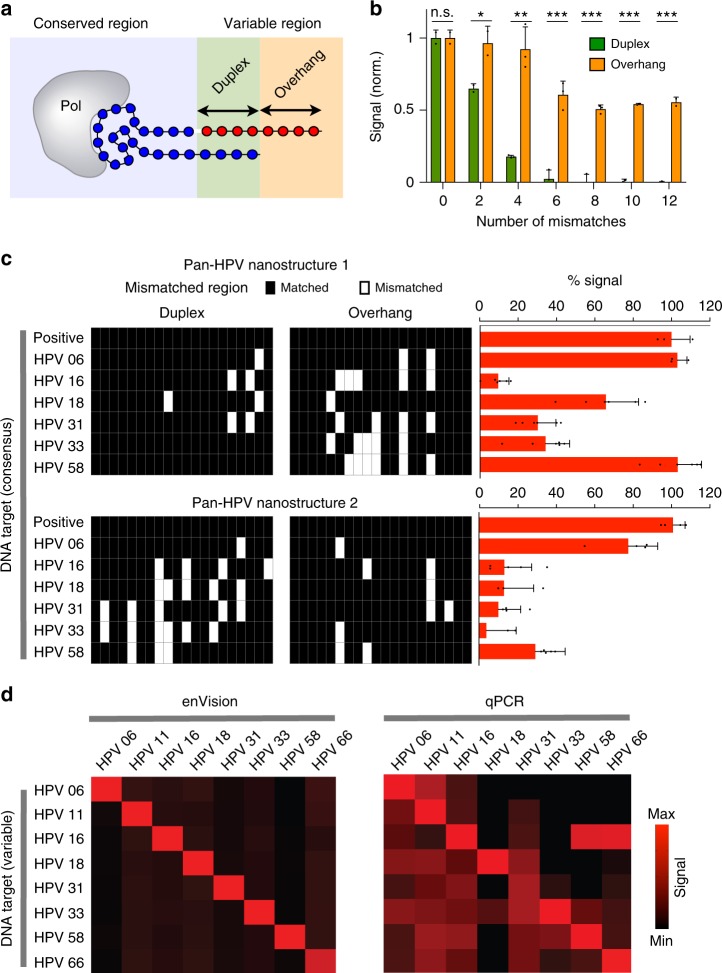
Programmability of enVision. **a** Schematic of the programmable recognition nanostructure. The hybrid structure consists of a conserved sequence region, that binds to inactivate DNA polymerase (pol), and a variable region (duplex and overhang segments) that can be made complementary to target DNA. Not drawn to scale. **b** Effects of target mismatches in the variable region. Synthetic DNA targets, designed to have varying numbers of mismatches against the variable region, were incubated with the recognition nanostructure. All signals were normalized against that of the complementary DNA target (0 mismatch). Mismatches against the duplex region produced significantly lower signals (*P < 0.05, ** *P* < 0.005, ***P < 0.0005, n.s. not significant, Student’s *t* test). **c** Pan-HPV recognition. Two pan-HPV recognition nanostructures were developed according to the HPV consensus genome, to harbor different numbers of mismatches against DNA targets obtained from six HPV subtypes. All mismatches were mapped to the duplex and overhang regions. Nanostructure 1, that accommodated more mismatches in the overhang region, demonstrated better pan-recognition capability. All signals were normalized against that of the complementary DNA target (positive). **d** Comparison of enVision and qPCR measurements for specific HPV subtyping. At a cutoff for 100% sensitivity, enVision had 100% specificity (56/56) while SYBR Green-based qPCR had 92.9% specificity (52/56). Specific nanostructures were designed according to a highly variable region of the HPV genome with sequence variations contained within the sensitive duplex region. DNA targets from different HPV subtypes were measured via color intensity through the enVision smartphone platform (left) and cycle counts through the SYBR-Green qPCR system (right). All signals were acquired relative to appropriate controls (i.e., water as a no-template control). Signals from respective detection systems were globally presented in the form of heat maps for comparisons of assay performance. All measurements were performed in triplicate, and the data are displayed as mean ± s.d. in **b** and **c**

To design assays for visual detection of nucleic acids, we investigated the programmability of the recognition nanostructure. The hybrid structure consists of a conserved sequence region that folds to bind and inhibit DNA polymerase, and a variable region (i.e., duplex and overhang segments) that can be made complementary to target DNA (Fig. 3a). By inducing mismatches between the variable region of the recognition nanostructure and the target DNA, we identified that the duplex region showed a higher sensitivity to sequence mismatches, as compared to the overhang region (Fig. 3b). We thus reasoned that the duplex region could confer strong sequence specificity, while the overhang region could accommodate more sequence variability, a feature useful for pan-detection.

We validated this design principle by developing two pan-HPV recognition nanostructures (Fig. 3c). The structures were designed by matching against the HPV consensus genome, and contained varying number of mismatches against DNA targets from six HPV subtypes (Supplementary Table 2). By mapping all the mismatched regions, we found that Pan-HPV Nanostructure 1 which accommodated more mismatches in the overhang region demonstrated better pan-detection capability of the consensus genome. Pan-HPV Nanostructure 2 which contained more mismatches in the duplex region showed a reduced signal in general, indicating the importance of the duplex region in conferring assay specificity.

Leveraging on this design principle, we next designed specific nanostructures for HPV subtyping. We identified a highly variable region of the HPV genome to design a new group of recognition nanostructures for specific differentiation of HPV subtypes (i.e., HPV 6, 11, 16, 18, 31, 33, 58, and 66) (Fig. 3d, left); sequence variability was designed to be contained within the sensitive duplex region. As compared to conventional qPCR analysis, which requires a pair of primers for target amplification and detection, the enVision recognition nanostructure could be easily adapted, as its specificity is determined by a single probe sequence (i.e., the variable region). We used this programmability to rapidly design new specific HPV subtyping nanostructures (Supplementary Table 2) and integrated them with the enVision technology. In a comparative analysis where we designed PCR primers to detect the same HPV DNA targets (Supplementary Table 3), the enVision platform demonstrated better specificity (100% specificity, 56/56, Fig. 3d, left), while the SYBR Green-based qPCR yielded more false positives (92.9% specificity, 52/56, Fig. 3d, right), likely due to non-specific amplification in the qPCR analysis (Supplementary Fig. 8). Importantly, due to differences in the sensing mechanisms (i.e., enVision detects via hybridization-activated signal enhancement and qPCR detects via nucleic acid target amplification), we further demonstrated that the enVision technology could directly distinguish specific HPV RNA targets, without needing any reverse transcription—a prerequisite for qPCR detection of RNA targets to ensure enzyme compatibility (Supplementary Fig. 9). All sequences used to transcribe the RNA targets can be found in Supplementary Table 3.

### Multiplexed assay for enhanced detection coverage

**Fig. 4:**
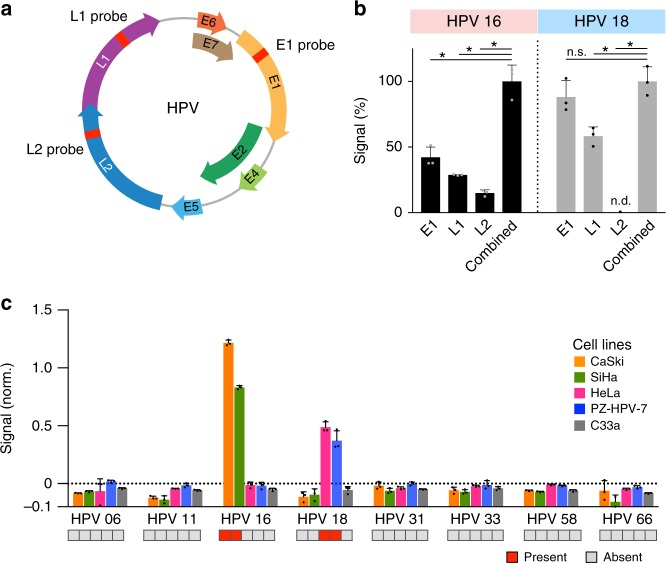
Multiplexed enVision for multi-loci coverage. **a** New probe loci in HPV genome map. The HPV genome is made up of seven early expressed (E) genes and two capsid protein (L) genes. New nanostructure recognition probes were designed for each HPV subtype to identify the *E1*, *L1*, and *L2* loci, respectively. **b** Multiplexed enVision assays for high-coverage, multi-loci detection. Genomic DNA obtained from CaSki cells (left, HPV 16-positive) and HeLa cells (right, HPV 18-positive) were incubated directly with individual recognition probes (*E1*, *L1*, and *L2*, respectively) or a pool of three probes (combined) to determine the cellular HPV infection status. The combined probes showed significantly higher signals as compared to any of the individualized assays (*P < 0.0005, n.s. not significant, Student’s *t* test; n.d. not detected). All signals were normalized as a percentage to their respective combined signals. **c** HPV subtyping in cell lines. Genomic DNA from cell lines were profiled directly using the multiplexed enVision assays for different HPV subtypes. The measurements correlated well with the known HPV infections of the cell lines, as reported by previous literatures (red: present, gray: absent). All measurements were performed in triplicate, and the data are displayed as mean ± s.d

To improve detection coverage, we next determined if multiplexed enVision assays could be performed simultaneously in a single chamber. While clinical HPV assays typically detect a single region of the viral genome (the *L1* locus) for infection determination, partial viral genome integrations into the host cells are also possible and can lead to cancerous growth^24,25^. Using non-conserved regions in the HPV viral genome, we thus designed various enVision recognition nanostructures to detect the *E1*, *L1*, and *L2* loci to improve the detection coverage (Fig. 4a; Supplementary Table 4). These modular recognition nanostructures could be readily configured into different logic gates (e.g., OR, AND, NOT, NAND, and NOR), in a single reaction chamber, to perform molecular computations and enable visual readouts from different combinations of DNA targets (Supplementary Fig. 10**;** Supplementary Table 5).

By mixing three types of locus-specific nanostructures (i.e., *E1*, *L1*, and *L2* loci) in a single reaction, we thus created the OR logic gate configuration to enhance detection coverage for each HPV subtype (e.g., HPV 16 and HPV 18). We tested this high-coverage, multi-loci HPV detection directly with genomic DNA of cervical cancer cell lines, which were independently validated with PCR analysis (Supplementary Fig. 11). Our data showed that the multiplexed enVision assay could further enhance the detection signal, as compared to individualized detection with the *E1*, *L1*, and *L2* recognition probes, respectively (Fig. 4b). Importantly, while true-positives showed significant signal enhancement, negative samples demonstrated minimal background signal change with this multiplexed approach (Supplementary Fig. 12).

Using the multiplexed enVision assays, we next performed HPV profiling in human cervical cancer cell lines (Fig. 4c). All experiments were performed at room temperature, directly from cellular genomic DNA, without any pre-amplification. The multiplexed enVision analysis not only improved the signal enhancement, but also expanded the detection coverage to accurately identify the cells’ infection status and subtypes, as compared to any single locus detection (Supplementary Fig. 12). Our resultant measurements correlated well with published literature studies^26–28^: CaSki and SiHa cells showed significant signals for HPV 16 only, while HeLa and PZ-HPV-7 showed positive signals for HPV 18 only.

We further benchmarked the enVision performance against another isothermal detection technique, namely LAMP, for HPV profiling of human cancer cell lines. On the same divergent regions of the HPV genome (Fig. 4a), we applied published criteria^29,30^ to design LAMP primer sets against *E1*, *L1* and *L2* loci, respectively, for each HPV subtype (see Methods for details). All LAMP sequence information can be found in Supplementary Table 6. In comparison to LAMP’s limited primer options, the enVision technology not only generated significantly more probe choices but also provided comprehensive coverage across all subtypes and loci tested (Supplementary Fig. 13a). We further compared the performance of the enVision technology against top-ranked LAMP primer sets, using genomic DNA isolated from cell lines of known infections (Supplementary Fig. 13b). Only the enVision technology showed accurate HPV subtyping (100% specificity and 83.3% sensitivity) while LAMP produced significant false positives (75.0% specificity and 50.0% sensitivity) (e.g., CaSki cells, HPV 18; HeLa cells, HPV 16).

### HPV profiling of clinical samples

To test the clinical utility of the enVision platform in detecting and subtyping HPV infections, we conducted a feasibility study. We aimed at addressing two questions: (1) how accurate is the enVision platform in detecting HPV infections, and (2) can improved assay coverage identify previously undetectable infections.

**Fig. 5:**
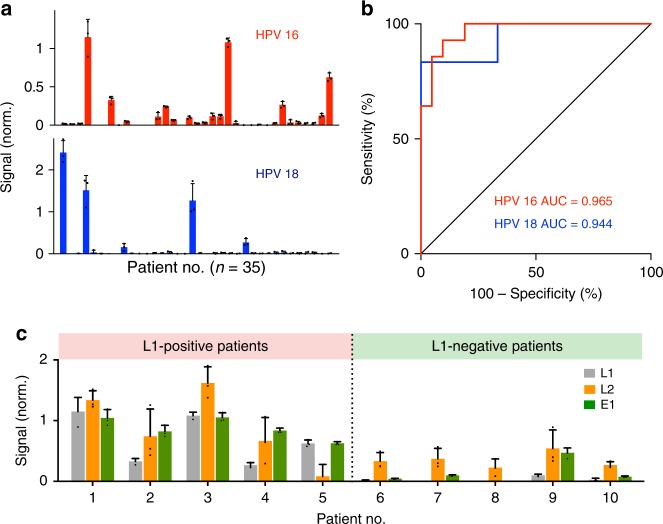
Molecular profiling of patient samples. **a** HPV 16 and HPV 18 signals were measured from clinical endocervical brush samples (*n* = 35). The *L1*-specific signals are shown for comparison with the clinical gold standard. See Supplementary Fig. 14 for multi-loci measurements on all clinical specimens. **b** Receiver operator characteristic (ROC) curves of the HPV 16 and HPV 18 *L1* locus assays were used to determine the detection accuracies. HPV 16 assay showed 92.9% sensitivity (13/14) and 90.5% specificity (19/21) and HPV 18 assay showed 83.3% sensitivity (5/6) and 100% specificity (29/29) at the Youden’s index cutoff. **c** Locus-specific HPV 16 enVision assays (*L1*, *L2*, and *E1* locus assays) were performed in all patients. Representative examples from *L1*-positive (left) and *L1*-negative (right) patients are shown. Note that in the subset of *L1*-negative patients, the inclusion of *L2* and *E1* locus assays could improve the detection coverage to identify previously undetectable infections. This was further validated through independent Taqman fluorescence analysis, which showed a high concordance with the enVision results in all tested clinical specimens (see Supplementary Fig. 15). All measurements were performed in triplicate, and the data are displayed as mean ± s.d. AUC, area under the curve

Using clinical endocervical brush samples, we performed the enVision assays to determine the HPV infection status. We acquired patient samples (*n* = 35) and used the enVision platform to measure HPV 16 and HPV 18 *L1* loci in patient genome (Fig. 5a), so as to compare directly against conventional gold standard (i.e., Cobas HPV which tests only for the *L1* locus through qPCR analysis^13^). The enVision platform achieved a high detection accuracy with the clinical reports (HPV 16, AUC = 0.965; HPV 18, AUC = 0.944) (Fig. 5b). The developed HPV 16 assay showed 92.9% sensitivity (13/14) and 90.5% specificity (19/21) and the HPV 18 assay showed 83.3% sensitivity (5/6) and 100% specificity (29/29), as measured by Youden’s index.

We also used the high-coverage multi-loci enVision assays to measure, in addition to the *L1* locus, *L2* and *E1* locus integrations in these clinical samples (Supplementary Fig. 14). In the HPV 16 *L1*-positive clinical samples, we continued to observe enhanced visual signals for the other detection loci. Interestingly, in a subset of the *L1*-negative samples, the high-coverage enVision system detected specific *L2* and/or *E1* locus integrations (Fig. 5c). We further validated this finding by designing an independent Taqman fluorescence analysis (Supplementary Table 4), which showed a high concordance with the enVision results in all tested clinical specimens (Supplementary Fig. 15). Our data thus indicated that the multiplexed enVision platform could improve the detection coverage to identify previously undetectable infections.

## Discussion

By integrating a series of enzyme-assisted DNA nanostructures, the enVision platform enables sensitive and versatile detection of diverse pathogen nucleic acids by the naked eye. In comparison to nucleic acid amplification techniques, the enVision detects via direct target hybridization and independent visual signal enhancement. The technology is thus well-suited for clinical applications: (1) the cascading signal enhancement produces a rapid and sensitive color readout for the naked eye upon target hybridization (without needing for target amplification). The entire assay can thus be performed at room temperature and readily quantified with smartphones; (2) the DNA nanostructures effectively decouple recognition and signaling functionalities to enable highly programmable detection and logic computation; and (3) the microfluidic integration complements the assay modularity and speeds up the reactions. In comparison to clinical gold standard and other isothermal detection technologies, the enVision platform not only demonstrated superior sensitivity and specificity, but also afforded versatile capabilities (e.g., direct RNA detection, logic computation and assay modularity) with minimal equipment requirement (Supplementary Table 7).

The enVision advantages are multifold. First, sensing mechanism. The technology activates immediately upon target hybridization to transduce significant signal enhancement. By incorporating a dual-enzyme signaling cascade (i.e., DNA polymerase and horseradish peroxidase HRP), enVision benefits from the activities of both enzymes and ameliorates each enzyme’s individual shortcomings for visual detection of diverse targets. For example, in comparison to traditional HRP enhancement, which is commonly used in enzyme-linked immunosorbent assays (ELISA) for protein detection, the enVision’s dual signaling cascade not only expands the target options to enable direct and visual detection of DNA and RNA targets, but also significantly improves the visual detection sensitivity through additional polymerase activity (>1000-fold better than sole HRP enhancement). In comparison to nucleic acid target amplification techniques, the enVision detects by direct nucleic acid hybridization to trigger an exponential visual signal enhancement. The visual enhancement is universal (i.e., target-independent); this improves the assay specificity by reducing target-dependent false positives, as commonly seen in polymerase-based target amplification techniques. Due to this sensing mechanism, the technology could be applied to detect different types of nucleic acids accurately and directly (e.g., DNA and RNA, no need for cDNA conversion), without requiring extensive equipment, to produce a rapid and sensitive color readout that is visible to the naked eye and quantifiable by smartphones.

Second, assay programmability. By decoupling recognition from signaling, the technology enables modular detection and versatile integration. New assays could be readily designed by modifying a single sequence region in the highly programmable recognition element alone and configured to perform various molecular computations. The technology can thus produce more probe choices, demonstrate more comprehensive target sequence coverage and achieve more specific and accurate detection. As the enVision recognition element consists of regular DNA strands (no chemical modifications on the DNA oligonucleotides), it is inexpensive to synthesize and develop new assays. The universal signaling element could be used for all visual measurements. When implemented on a configurable microfluidic platform, the enVision technology thus shows a high detection sensitivity and versatility, to enable visual profiling of diverse pathogen nucleic acids from infected cells at room temperature. Through multi-loci multiplexing in a single reaction, we further developed a high-coverage enVision system to interrogate various virus–host genome integration loci. Using HPV as a clinical model, we demonstrate the technology’s clinical utility for molecular-typing of infections in patient endocervical samples, by improving inter-subtype differentiation as well as intra-subtype detection coverage.

The scientific and clinical applications of the developed technology are potentially broad. The recognition nanostructure can be easily adapted, at a very low cost, to detect different target sequences with similar efficiencies (i.e., only a single probe sequence composed of regular nucleotides needs to be adapted, instead of requiring new sets of conventional primers or dedicated chemically modified reporters). The signaling nanostructure is universal and can be used for all target sequences. Such modularity not only eases new assay development, but also enables programmable configuration to perform molecular computations. We thus anticipate that the technology will be particularly useful for investigating rapid viral mutations that escape immune detection (i.e., RNA viruses)^31^ as well as multiple virus–host genome integrations^32^. Clinically, the enVision technology could be deployed to provide new visual assays for emerging infectious diseases (e.g., Zika and Ebola)^9^ or enable easy detection of various high-prevalence infections (e.g., hepatitis and dengue) at the patient level. Its visual detection and low equipment requirement make the technology well-suited for point-of-care detection workflow in community clinics and resource-limited settings.

We further anticipate that several technical modifications could be made to enhance the current technology. First, the present fluidics (i.e., four assay cassettes) could be readily revised to accommodate more and smaller chambers. As the current nucleic acid processing is performed off-chip, future work could integrate additional sample preparation modules to enable nucleic acid extraction and treatment, thereby enhancing the platform’s clinical utility^23^. Such a new design would enable practical, array-type visual detection of multiple pathogen nucleic acids. Second, in this current detection platform, we used smartphones and a corrected lighting system to image and analyze the visual readouts. Since smartphones are becoming ubiquitous and possess more analytical capabilities^8,33,34^, we foresee that further image correction and analysis algorithms could be implemented directly in smartphones to automatically correct for lighting differences and quantify color intensities for real-world applications. Finally, large cohort studies on the detection of pathogen nucleic acids across a spectrum of diseases (e.g., other infections, cancers, inflammatory disorders) using various biological specimens (e.g., tissue, blood, urine) could be performed to validate the clinical utility of the enVision technology for diverse visual detection.

## Methods

### Recognition nanostructure characterization

All sequences can be found in the Supplementary Tables and were purchased from Integrated DNA Technologies (IDT). To prepare the recognition nanostructure, we mixed an equal molar ratio of DNA aptamer and inverter oligonucleotide in a buffer of 50 mM NaCl, 1.5 mM MgCl_2_, and 50 mM Tris–HCl buffer (pH 8.5). The mixture was incubated at 95 °C for 5 min and slowly cooled at 0.1 °C/s until the reaction reached 25 °C, before the addition of Taq DNA polymerase (GoTaq, Promega) to form the hybrid complex. To characterize the assembly and activity of the recognition nanostructure in the presence of DNA targets, varying concentrations of target oligonucleotides were added to this mixture. Real-time association and dissociation kinetics of the complex were measured by bio-layer interferometry (Pall Fortebio). Briefly, pre-assembled biotin-aptamer was immobilized onto streptavidin-functionalized interferometry sensor. After a brief washing step, Taq polymerase was added; this was followed by an incubation with target oligonucleotides. All binding data (changes in optical thickness of the biolayer) were measured as wavelength shifts, in a continuous manner. We further measured the polymerase activity through 5′ exonuclease degradation of Taqman probes (Applied Biosystems). Fluorescence readings were taken every 2 min, in the presence of the Taqman probe, and normalized as described (see Data normalization below) to compare the resultant polymerase activity.

### Signaling nanostructure characterization

Stability of the signaling nanostructure, as compared to that of a similarly sized linear template, was determined in two different ways. First, we annealed both reactions (i.e., nanostructure and linear template) at room temperature, and analyzed the primed vs. unprimed fractions in each reaction through gel electrophoresis. Second, a melting curve analysis was performed by mixing the oligonucleotides with 10,000× diluted SYBR Green I dye (Invitrogen), heating the reaction to 90 °C, fast cooling to 45 °C at 1.6 °C/s, and slow heating to 90 °C at 0.075 °C/s while visualizing the intercalated fluorescence intensity (Applied Biosystems). We further determined the ability of the signaling nanostructure to improve polymerase activity through Taqman analysis. The polymerase activity was determined at different nucleotide positions away from the starting primed sites, through 5′ exonuclease degradation of differentially placed Taqman probes (Applied Biosystems).

### enVision device fabrication

A prototype enVision device comprising 4 assay cassettes and a common cartridge was fabricated from polydimethylsiloxane (PDMS, Dow Corning) and poly(methyl methacrylate) (PMMA) respectively. Each assay cassette was fabricated by plasma bonding two layers of PDMS pieces together (50 mTorr, 50 W, 1 min). The 200 μm-thick cast molds were prepared via conventional photolithography using SU-8 photoresist and silicon wafers. Reaction chambers and microchannels were replicated by pouring uncured PDMS (10:1 elastomer base to curing agent ratio) onto the cast molds. After polymer curing (75 °C for 30 min), the two PDMS pieces were assembled together. The common cartridge was fabricated by CO_2_ laser ablation (Universal Laser Systems). Four polycarbonate membranes with pore size of 0.2 μm (Avanti Polar Lipids) were embedded between the two PMMA layers thermally bonded together (125 °C for 30 min). A NanoPort assembly (Upchurch Scientific) was adhered to the outlet on the common cartridge to allow for fluidic connection.

### Signaling nanostructure immobilization

We used amine-modified oligonucleotides (IDT) for functionalization. To immobilize the oligonucleotides onto the microfluidic device, we used polystyrene beads to increase the surface area for anchoring the oligonucleotides. Approximately 3 μm carboxylic acid-modified polystyrene beads (Spherotech) were washed in PBS buffer before being resuspended in MES buffer (Thermo Fisher Scientific). The beads were activated with EDC/sulfo-NHS (Pierce Biotechnology) for 15 min at room temperature, and incubated with excess 5′ amine-modified oligonucleotides in PBS buffer at room temperature for 2 h. The beads were then washed through centrifugation and resuspended in 10 mM Tris-EDTA buffer.

### Device preparation

To prepare the device for operation, we lyophilized the bead-immobilized signaling nanostructures as well as the modified dNTP reaction mixture onto the common cartridge. All devices were flushed with ethanol and PBS buffer before lyophilization. Briefly, a 4 mM stock dNTP reaction mixture was prepared by mixing dATP, dGTP, dTTP, and 25% dCTP: 75% biotin-16-aminoallyl-2′-dCTP (Trilink Biotechnologies). In total 50 µg of DNA-functionalized polystyrene beads (3 μm, prepared as above) were introduced in 20 µl of dNTP mixture onto the device polycarbonate membranes, and allowed to dry under vacuum for 30 min (Labconco FreeZone).

### Operation of enVision platform

Operation steps are illustrated in Supplementary Fig. 2. Nucleic acid samples were added to the inlets of individual assay cassettes, each preloaded with assembled recognition nanostructures, in a buffer containing 50 mM NaCl, 1.5 mM MgCl_2_, and 50 mM Tris–HCl at pH 8.5. The assay cassettes were then mounted onto the common signaling cartridge, prepared as described above. For the purpose of assay optimization, we used a syringe pump (Harvard Instruments) to exert a negative pressure at the common cartridge outlet to actuate parallel fluidic movement in all four assay cassettes. Each reaction mix would pass through a serpentine channel for effective mixing and polymerase activation (10 µl/min, 1 min), before entering into a reaction chamber. The solution was then incubated for 20 min in the presence of DNA-functionalized polystyrene beads and dNTP reaction mixture. The beads were flushed in excess PBS buffer (10 µl/min, 3 min), before incubation with streptavidin-HRP (BD Biosciences, 3 min) and 3,3′-diaminobenzidine substate (DAB, Thermo Fisher Scientific, 3 min).

As the crucial steps in the enVision workflow were largely incubation and washing, where precise flow rates become unnecessary, we could thus use a withdrawal septum (Thermo Fisher) to actuate fluid movement through negative pressure. All reactions were completed at room temperature, and included appropriate negative controls. Visual readouts of samples and controls were imaged directly using a mobile smartphone (Samsung). Color images were converted to greyscale, and the average (mean) black pixel intensity of each membrane area was used for numerical quantification of the signal.

### Data normalization

$$\tilde{I}(target,t_i) = \frac{{I(target,t_i) - I(target,{\it{t}}_0)}}{{I(control,t_i) - I(control,t_0)}} - 1$$

where $\widetilde I(target,t_i)$ = normalized signal intensity for target at a given time point (*t*_*i*_); *I* (target or control, *t*_*i*_) = raw signal intensity for target or control at a given time point (*t*_*i*_); *I* (target or control, *t*_*o*_) = raw signal intensity for target or control at time 0 (*t*_*o*_) We calculated the signal intensity difference (visual color or fluorescence) of a sample as the difference in intensity taken at a given time point (*t*_*i*_) and its initial intensity (*t*_*o*_). This value was then normalized to that of the control sample (no-target control) that was run concurrently. $\tilde I(control,t_i)$ was scaled to 0 for data representation.

### Sequence design

HPV genome sequences were obtained from GenBank through the following reference numbers (HPV 6: AF092932.1, HPV 11: FR872717.1, HPV 16: K02718.1, HPV 18: AY262282.1, HPV 31: J04353.1, HPV 33: M12732.1, HPV 58: D90400.1, HPV 66: U31794.1). Multiple sequence alignment was performed using Clustal Omega^35^. From the resulting sequence alignment, we defined highly conserved and divergent sequences to design the pan-recognition and subtype-specific recognition nanostructures, respectively. Briefly, we identified highly conserved regions (20 bp, <3 non-identical base pairs) which were flanked immediately upstream or downstream by less conserved regions (20 bp, <6 non-identical base pairs). We selected the most conserved motifs to form the recognition domain of our pan-HPV recognition nanostructures. To design subtype-specific recognition nanostructures, we located highly divergent regions (40 bp, <12 identical base pairs). We used a similar approach to define divergent regions in the *L1*, *L2*, and *E1* genes of the HPV genome, and designed locus-specific recognition nanostructures for each HPV subtype to improve the detection coverage. All sequences designed can be found in Supplementary Tables.

### Mismatch characterization

To evaluate the mismatch sensitivity of the duplex and overhang region in the recognition nanostructure, we randomly mutated base pairs at every two-nucleotide interval in the complementary DNA targets and used the enVision platform to measure the resultant signal changes. To determine the pan-HPV detection capabilities of the recognition nanostructures, we used sequences of six HPV subtypes (HPV 6, 16, 18, 31, 33, and 58) as well as a fully complementary sequence (positive control) as the target sequences. We mapped the mismatched nucleotides to the duplex and overhang regions respectively and measured the resulted signal changes.

### Asymmetric amplification

In the case of minuscule amounts of samples, we prepared single-stranded DNA through a nested asymmetric PCR amplification. The amplification could be accomplished in <1 h. For the exponential amplification, we used 0.8 µM of forward and reverse primers (IDT), and 5 units of GoTaq DNA polymerase (Promega) in 1× GoTaq buffer containing 2.5 mM dNTPs. This was followed by a linear asymmetric PCR amplification, where we used excess reverse primer only, in the presence of GoTaq DNA polymerase (Promega) in 1× GoTaq buffer containing 2.5 mM dNTPs. The following thermocycling conditions were used for the entire processing: 95 °C for 5 min, 35 cycles of 95 °C for 30 s and 52 °C for 60 s, and a final 4 °C holding step. The PCR solution was used directly for all subsequent measurements.

### Isothermal asymmetric amplification

To achieve isothermal asymmetric amplification, we adopted nucleic acid sequence based amplification (NASBA). The entire process could be completed in <1 h. We used 4 µM of each of the forward and reverse primers (IDT), 80 units of T7 RNA polymerase, 1 unit of RNase H, 80 units of GoScript Reverse Transcriptase, and 1 unit of RNase Inhibitor (Promega) in 1× transcription optimized buffer containing 2.5 mM dNTPs, 5 mM NTPs, 6 mM MgCl_2_, 5 mM DTT, and 15% DMSO. Upon sample addition, the mixture was maintained at 37 °C for 45 min, before being heated to 75 °C to halt the reaction.

### SYBR qPCR comparison

We used the HPV subtype-specific sequences to perform this comparison. Subtype-specific detection of synthetic oligonucleotides was carried out on the enVision platform as described above. For qPCR experiments (Applied Biosystems), we designed specific PCR primer pairs to span the identical target sequences for each HPV subtype. Briefly, 100 fmol of synthetic template was mixed with 5 units of GoTaq DNA polymerase (Promega) in 1× GoTaq buffer containing 2.5 mM dNTPs and 0.8 µM forward and reverse primers, using the following thermal cycling protocol: 50 °C for 2 min, 95 °C for 10 min, 40 cycles of 95 °C for 15 s and 48 °C for 1 min. All qPCR analyses were followed by a melting curve step ramping from 50 to 95 °C at a ramp rate of 0.075 °C/s to determine any nonspecific amplification. A no-template control (water) was included for every primer assay to ensure that no primer dimers were formed in the qPCR reaction. We determined the qPCR threshold cycle (Ct) signal for each primer pair/template combination. The signal produced by each qPCR primer assay was normalized across all targets tested for the assay to determine the assay specificity.

### Direct RNA detection

We prepared RNA targets via T7 RNA Polymerase (New England Biolabs). Briefly, annealed DNA template with T7 promoter primer (Supplemental Table 3) (1 pmol) was incubated with T7 RNA Polymerase in 1× buffer at 37 °C for 2 h, before the addition of 2 units of RNase-Free DNase I (Promega) and incubation at 41 °C for 20 min. The produced RNA was precipitated in 0.3 M sodium acetate. This mixture was treated with 1 volume of isopropanol and incubated at 4 °C for 1 h, before being centrifuged (12,000 × *g* at 4 °C for 30 min). The pellet was washed twice in chilled ethanol (75%), centrifuged as before, and air-dried at room temperature. When dissolved in nuclease-free water, the final RNA concentration was determined through absorbance measurements (Nanodrop, Fisher Scientific). For direct RNA detection, we added the prepared RNA targets, in the presence of 1 unit of SUPERase RNase Inhibitor (Thermo Fisher), to the enVision system. All detection and data normalization were performed as described above.

### Constructing enVision logic gates

All logic gate configurations and components were summarized in Supplementary Fig. 10 and Supplementary Table 5, respectively. Briefly, we used pre-assembled recognition nanostructures to demonstrate the AND, OR, NOT, NAND, and NOR functions. In constructing the different gates, we varied the combinations of recognition nanostructures used as well as the ratio of components in each hybrid nanostructure (i.e., DNA aptamer: DNA inverter: Taq polymerase) to program distinct computational functions. Specifically, to prepare the AND gate, two recognition nanostructures were used and each had its components mixed at the ratio (1: 1: 0.5); the OR gate—two structures (1: 1: 1); the NOT gate—one structure (1: 0: 1); the NAND gate—two structures (1: 0: 1); and NOR gate—two structures (1: 0: 0.5). All recognition nanostructures were pre-assembled, as described previously. The universal signaling nanostructure was used commonly in all logic gates. We tested the gate configurations with different combinations of DNA targets, as described in the truth tables, and compared the enVision signals with the expected outputs. Signals were normalized as described above. Normalized signals above the detection threshold (i.e., >3× s.d. of the background signal) were considered as true signals; otherwise a false signal was called.

### Cell culture and DNA extraction

All human cell lines were obtained from American Type Culture Collection; CaSki (ATCC CRL-1550), SiHa (ATCC HTB-35), HeLa (ATCC CCL-2), PZ-HPV-7 (ATCC CRL2221). CaSki cells were grown in RMPI-1640. SiHa, HeLa, and C33-a in Eagle’s Minimal Essential Medium, supplemented with 10% FBS and penicillin-streptomycin (Corning). PZ-HPV-7 in Keratinocyte Serum Free Medium, supplemented with bovine pituitary extract and human recombinant epidermal growth factor (ATCC). All cell lines were tested and free of mycoplasma contamination (MycoAlert Mycoplasma Detection Kit, Lonza). Genomic DNA was extracted from 80 to 90% confluent cell culture using DNeasy Blood & Tissue Kit (Qiagen) following the manufacturer’s recommended protocol.

### Comparison with LAMP

On the same divergent regions of the HPV genome, identified in the section of ‘sequence design’, we used the automated online LAMP primer design server (PrimerExplorer.jp) with the default settings^29^ to identify possible LAMP amplification primers. We compared the number of LAMP primer sets, as returned by the software, to the number of enVision recognition nanostructures that could be designed in the same region. For subsequent functionality test, we further compared the performance of the top-ranked LAMP primer sets with the enVision system. For each LAMP amplification, we used 0.2 µM of each primer in the primer set and 100 ng of purified genomic DNA extracted from cell lines of known HPV infections. This mixture was incubated with 8 units of Bst 2.0 DNA polymerase (New England Biolabs) in 1× isothermal amplification buffer II, 6 mM MgSO_4_, 2.5 mM dNTPs, at 70 °C for 1 h. The amplification product was purified via PCR cleanup spin columns (New England Biolabs) and quantified through gel electrophoresis.

### Clinical sample processing

The study was approved by the NUH and NUS Institutional Review Board (2016/01201). Endocervcial brush samples, fixed in BD SurePath^TM^ liquid-based Pap test solution, were collected from 35 individuals. We used the QIAamp DNA FFPE Tissue Kit (Qiagen) with a modified protocol to extract DNA from these clinical samples. Briefly, the samples were mixed with 2.5 ml sterile PBS buffer, 2.5 ml buffer ATL, and 80 µl Proteinase K to cover the brushes, and incubated at 56 °C. Following this incubation, the liquid was collected and incubated at 90 °C for 1 h. 3.5 ml buffer AL and 3.5 ml 100% ethanol was added to the sample. This mixture was passed through the extraction column, and washed sequentially with 500 µl of buffer AW1 and buffer AW2. The column was finally dried through centrifugation and the DNA sample was eluted in 50 µl of buffer ATE. The extracted genomic DNA was used directly in the assay, as described in “operation of enVision platform” or processed as described in “asymmetric amplification”, only in cases of residual leftover clinical samples. For all clinical samples, we determined the quality of the extracted DNA through Taqman PCR analysis of GAPDH housekeeping gene (Applied Biosystems) and all samples passed the quality check. All measurements on clinical samples were performed in an anonymized and blinded fashion and finalized before comparison with clinical reports (i.e., Cobas HPV).

### Gel electrophoresis

DNA samples were diluted with appropriate amounts of 6× loading dye (Thermo Fisher) and run on an 8% polyacrylamide gel with TAE buffer (Thermo Scientific) at 100 V. The gel was stained with SYBR Green I at 10,000× dilution in TAE buffer for 5 min before being visualized in a gel imager (Bio-Rad). Intensity and area (volume) of the resulting bands were calculated using ImageLab’s in-built algorithms (Bio-Rad).

### Statistical analysis

All measurements were performed in triplicates, and the data displayed as mean ± standard deviation. All significance tests were performed via a two-tailed Student’s *t* test. For inter-sample comparisons, multiple pairs of samples were each tested, and the resulting *P* values were adjusted for multiple hypothesis testing using Bonferroni correction. Values that had an adjusted *P* < 0.05 were determined as significant. Receiver operating characteristic (ROC) curves for the clinical study were generated from patient profiling data and constructed by plotting sensitivity versus (1–specificity), and the values of area under the curve (AUC) were computed using the trapezoidal rule. We used the clinical reports (i.e., Cobas HPV) as classifiers (true positives and true negatives). The optimal threshold for each marker was established from the point closest to the top-left part (perfect sensitivity or specificity) of the corresponding ROC curve. Detection sensitivity, specificity and accuracy were calculated using standard formulas. Statistical analysis was performed using the R-package (version 3.4.2).

### Data availability

Data supporting the findings of this study are available from the corresponding author on reasonable request.

## Electronic supplementary material

Supplementary Information
