# Supplementary material for: Visual and modular detection of pathogen nucleic acids with enzyme–DNA molecular complexes
Source: Nat Commun. 2018 Aug 13;9:3238. doi: 10.1038/s41467-018-05733-0 (PMC6089962; doi:10.1038/s41467-018-05733-0)
Supplement: Supplementary file 1 — Supplementary Information [file 41467_2018_5733_MOESM1_ESM.pdf]

SUPPLEMENTARY INFORMATION

Visual and modular detection of pathogen nucleic acids with enzyme-DNA molecular complexes

Nicholas R.Y. Ho[1,2,#], Geok Soon Lim[1,2,#], Noah R. Sundah[1,3], Diana Lim[4], Tze Ping Loh[1,5], Huilin Shao[1,2,3,6,*]

[1] Biomedical Institute for Global Health Research and Technology, National University of Singapore, Singapore 117599
[2] Institute of Molecular and Cell Biology, Agency for Science, Technology and Research, Singapore 138673
[3] Department of Biomedical Engineering, Faculty of Engineering, National University of Singapore, Singapore 117583
[4] Department of Pathology, National University Hospital, Singapore 119074
[5] Department of Laboratory Medicine, National University Hospital, Singapore 117599
[6] Department of Surgery, Yong Loo Lin School of Medicine, National University of Singapore, Singapore 119228
[#] These authors contributed equally

* Corresponding author
Huilin Shao, PhD
National University of Singapore
MD6, 14 Medical Drive
#14-01, Singapore 117599
(65) 6601 5885
huilin.shao@nus.edu.sg

SUPPLEMENTARY FIGURES

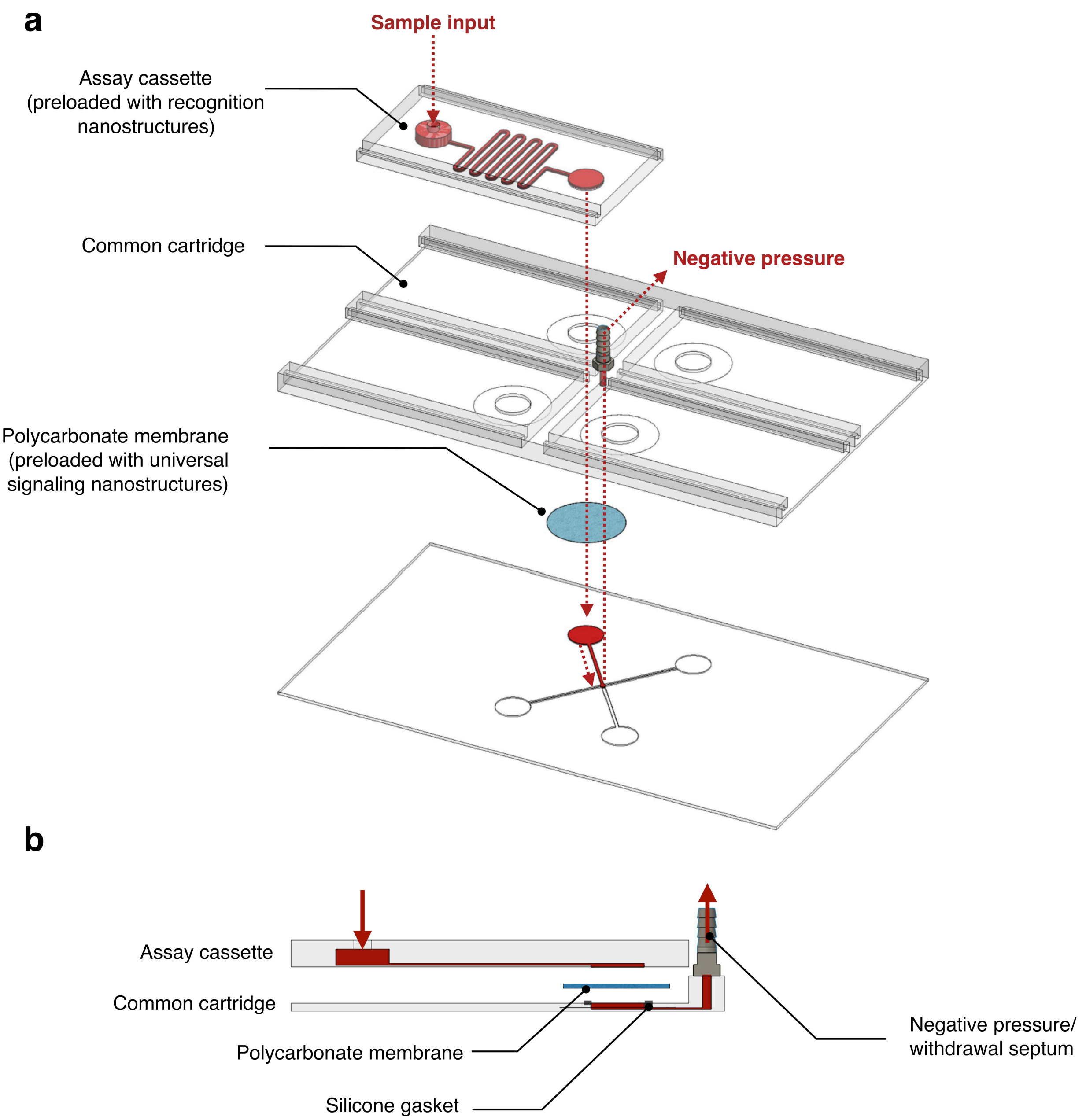

Supplementary Figure 1. Schematics of the enVision microfluidic platform. (a) Exploded schematic and **(b)** sectional side view of the platform. The platform comprises unique assay cassettes and a common signaling cartridge. Each assay cassette is preloaded with unique recognition nanostructures and contains a serpentine microchannel to improve mixing. Polycarbonate membranes are embedded in the common cartridge to immobilize the signaling nanostructures. Fluidic flow from the sample inlet to the common outlet, actuated by withdrawal septum, is indicated in red.

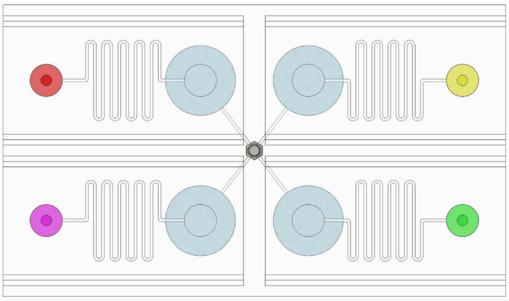

Step 1: Sample loading

Samples are added to the inlets of individual assay cassettes, each preloaded with recognition nanostructures targeting for specific DNA sequences.

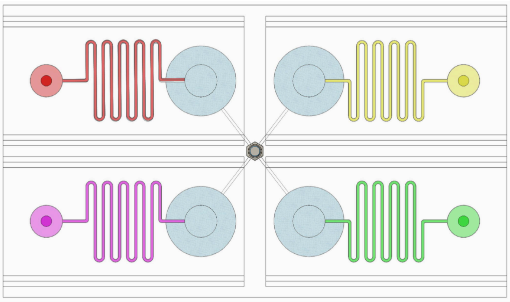

Step 2: Recognition

The assay cassettes are mounted onto the common cartridge. A negative pressure is used to actuate parallel fluid flow. Diffusive mixing in the serpentine channel improves sequence recognition and activation of the inactive polymerase if target is present.

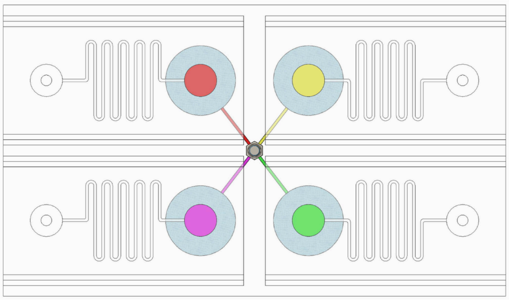

Step 3: Target-independent signal enhancement

The active polymerases are transferred into the reaction chambers, where they add HRP to the immobilized universal signaling nanostructures through biotinylated dNTPs.

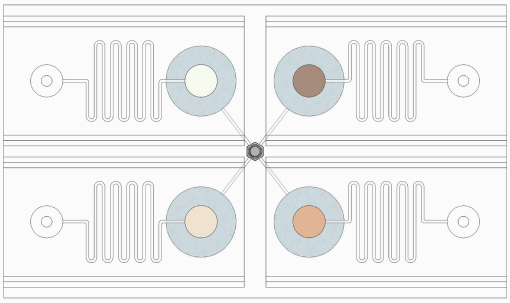

Step 4: Visual detection

Unbound HRP is removed and HRP substrate is introduced uniformly into the reaction chambers. Development of the HRP substrates leads to direct visual readout.

Legend:

Different enVision assays with unique recognition nanostructures

Supplementary Figure 2. Device operation.

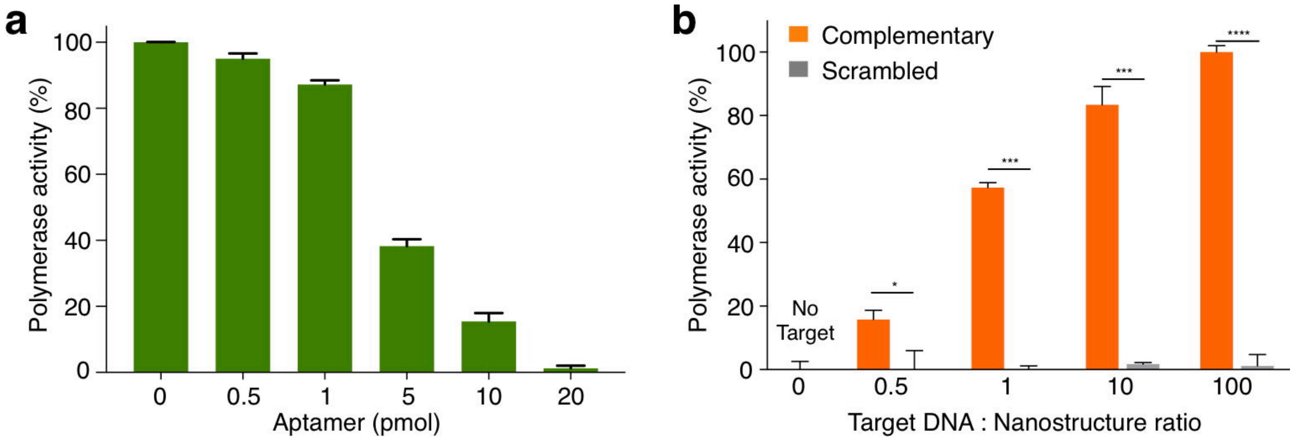

Supplementary Figure 3. Activity of the recognition nanostructure. (a) A varying amount of inhibitory aptamer was added to a fixed amount of polymerase (5 units) to determine the optimal ratio to complex the recognition nanostructure while maximizing the inhibitory effect. **(b)** To the optimized nanostructure complex, we incubated different amounts of complementary DNA target as well as scrambled oligonucleotide sequence as a control. Note that only complementary target resulted in strong and proportional increase in polymerase activity, while the scrambled oligonucleotide sequence produced negligible activity (*$P < 0.05$, ***$P < 0.0005$, **** $P < 0.00005$, Student's t test). All measurements were performed in triplicate, and the data are displayed as mean ± s.d.

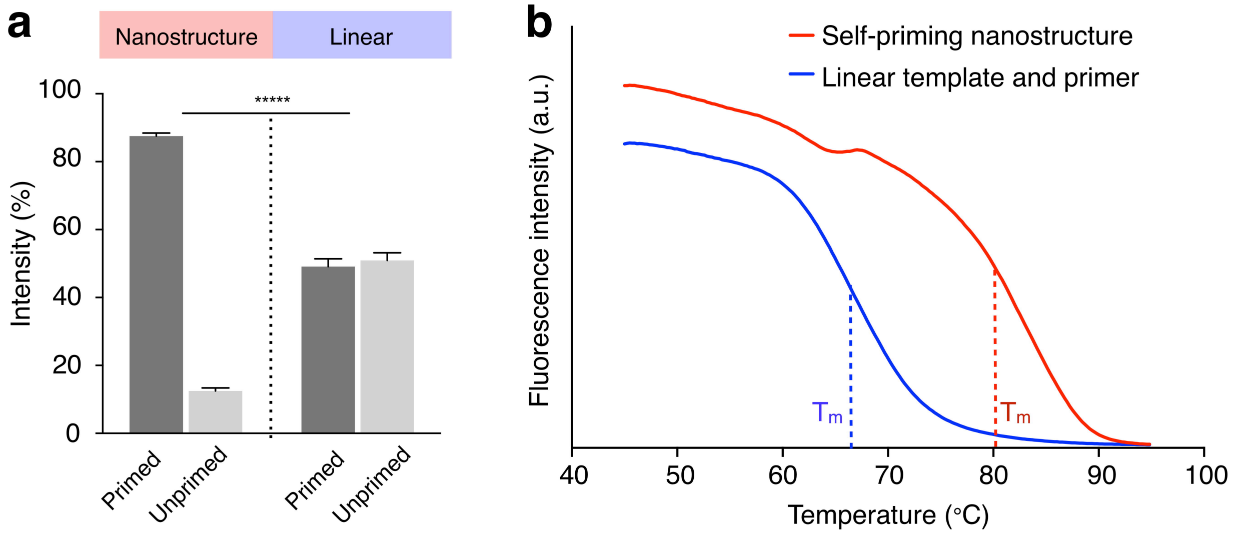

Supplementary Figure 4. Annealing of the signaling nanostructure. (a) The hairpin signaling nanostructure and its equivalent-sized linear counterpart (with excess primers) were resolved through 8 % native gel electrophoresis at room temperature. The band intensities of the primed and unprimed fractions in each sample were analyzed (***** $P < 0.000005$, Student's t test). **(b)** Melting curve analysis of the self-priming signaling nanostructure and its linear counterpart. SYBR green fluorescence intensities were recorded with increasing reaction temperature to assess the dissociation characteristics of double-stranded DNA. Dotted lines indicate the observed melting temperatures (T_m) of the respective nucleic acids. The nanostructure showed a higher T_m as compared to its linear counterpart (with excess primers). All measurements were performed in triplicate, and the data are displayed as mean ± s.d in **a**.

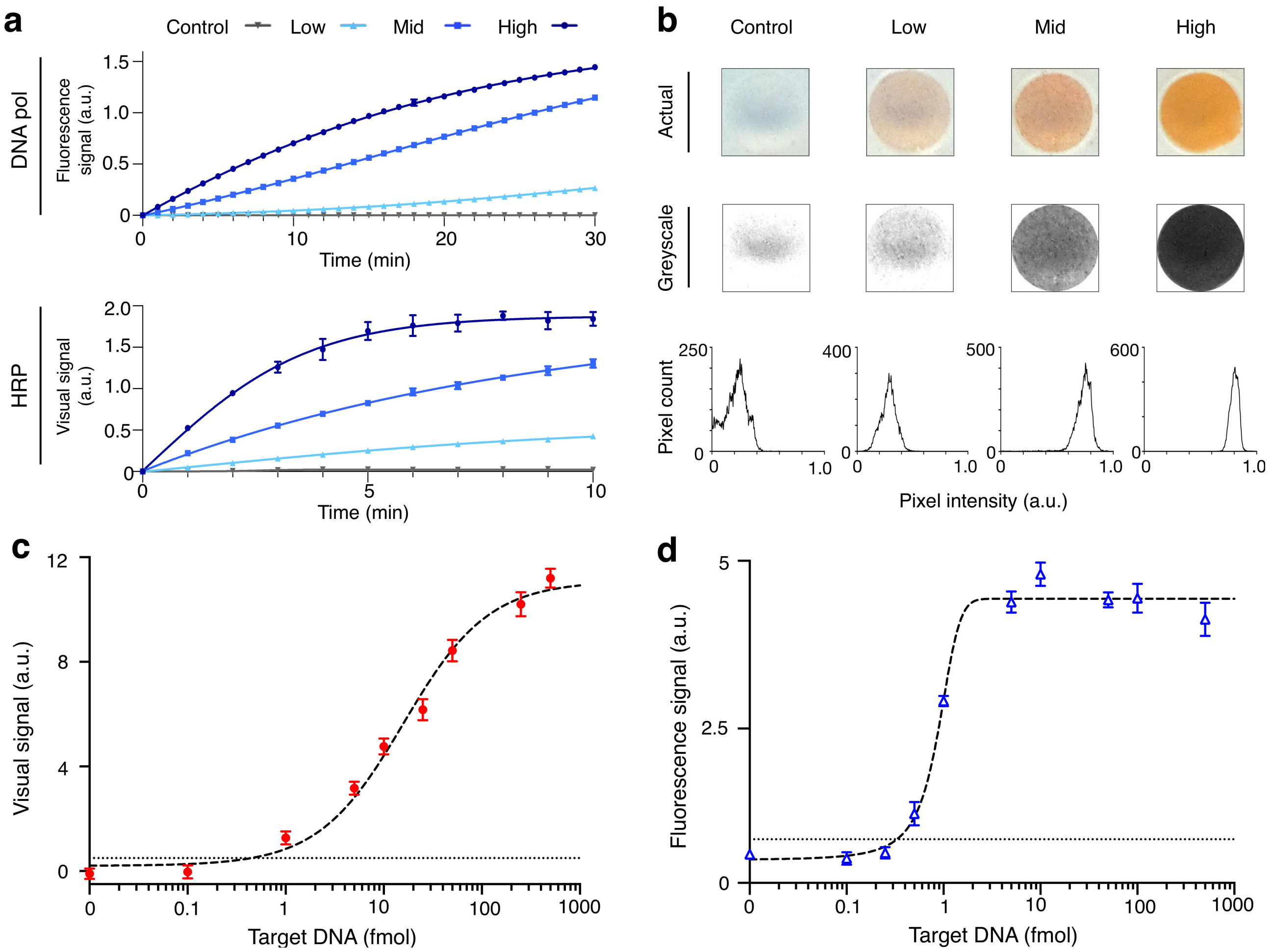

Supplementary Figure 5: Visual and fluorescence readouts. (a) Optimization of enzyme reactions**.** We measured the real-time activities of DNA polymerase (DNA pol, top) and HRP (bottom), in the presence of control (water) and varying amounts of DNA targets. Polymerase activity was determined via a Taqman assay (fluorescence measurement of 5' exonuclease degradation of Taqman probes), while HRP activity was determined via smartphone intensity measurement. Polymerase activity corresponds to Steps 1-3 in the device operation and HRP activity corresponds to Step 4 (see Supplementary Fig. 2 for more details). The optimized durations for these enzyme reactions were thus determined at ~ 20 min and 3 min, respectively. **(b)** Example images of enVision readouts (top), after image conversion to greyscale (middle) and the distribution of greyscale pixel intensities (bottom). The mean pixel intensity of each spot image was used for signal quantification and normalization. **(c)** Visual detection sensitivity of the enVision system. The detection limit (dotted line) was determined by directly titrating a known amount of target DNA (without asymmetric amplification) and measuring their associated visual signals through the enVision platform at room temperature. All visual signals were acquired through a smartphone. **(d)** Fluorescence detection sensitivity. The detection limit (dotted line) was determined by directly titrating a known amount of target DNA with the recognition nanostructure. The polymerase activity was measured via its 5' exonuclease degradation of fluorescent Taqman probes. All fluorescence signals were acquired though a commercial qPCR fluorescence detector. All measurements were performed in triplicate, and the data are displayed as mean ± s.d. a.u., arbitrary unit.

a

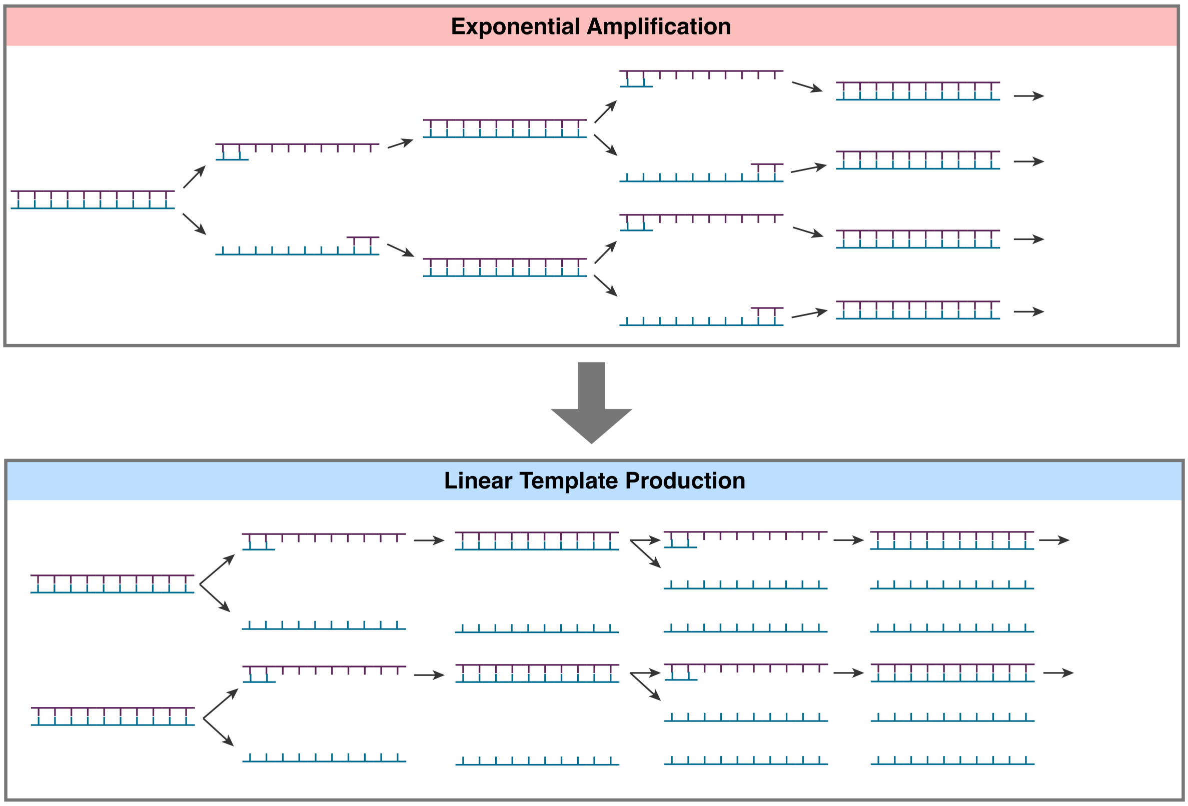

b

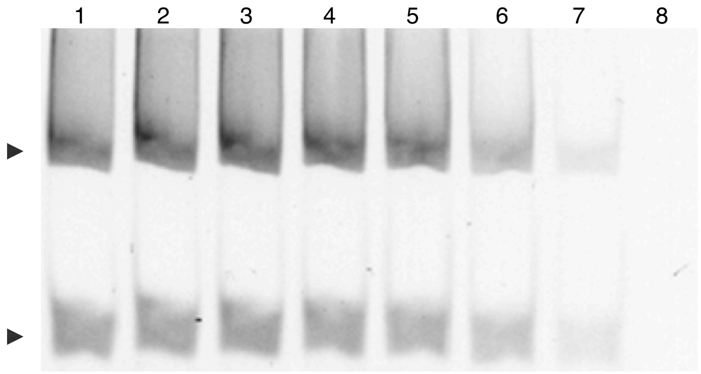

Supplementary Figure 6. Nested asymmetric amplification. (a) Schematic of the nested amplification. To significantly expand the population of single-stranded DNA for minuscule amounts of samples, a nested asymmetric PCR amplification was employed. The samples were first exponentially amplified, in the presence of equally concentrated dual primers, and subsequently linearly amplified, using an excess of a single primer. **(b)** Efficiency of the nested asymmetric amplification. Amplification products from (1) 1 pmole, (2) 100 fmole, (3) 10 fmole, (4) 1 fmole, (5) 100 amole, (6) 10 amole, (7) 1 amole, and (8) no template control of synthetic HPV16 sequence were analyzed on a 8 % PAGE gel. The upper arrow indicates larger, double-stranded products while the lower arrow corresponds to single-stranded products.

a

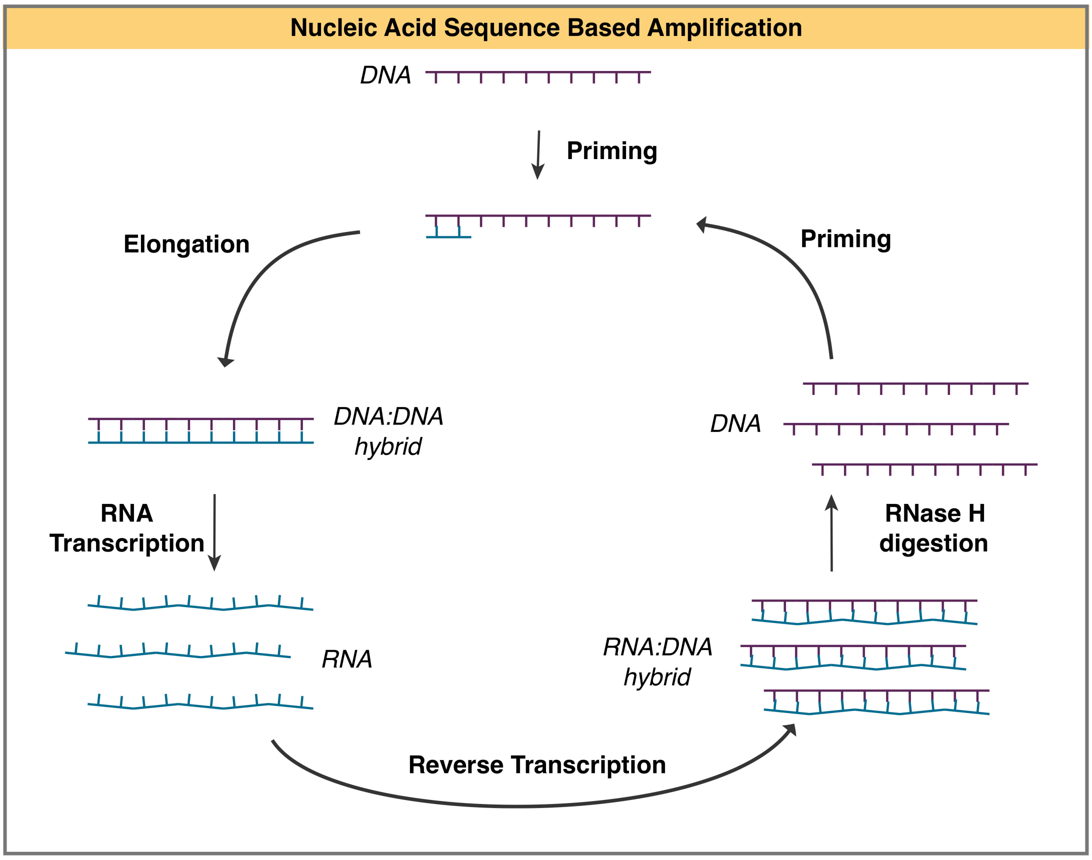

b

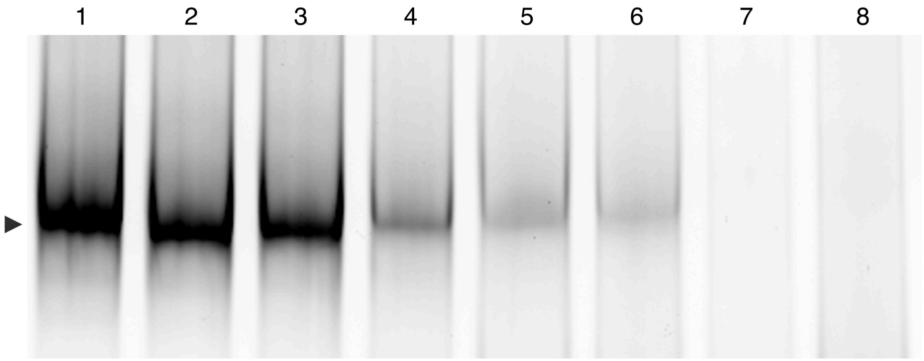

Supplementary Figure 7. Nucleic acid sequence based amplification (NASBA). (a) Schematic of NASBA. DNA targets were first primed for RNA transcription and single-stranded RNA amplification via the T7 RNA polymerase. The RNA products were then reverse transcribed into cDNA before being subjected to RNA digestion to produce single-stranded DNA products. **(b)** Efficiency of NASBA. Amplification products from (1) 1 pmole, (2) 100 fmole, (3) 10 fmole, (4) 1 fmole, (5) 100 amole, (6) 10 amole, (7) 1 amole, and (8) no template control of synthetic HPV 16 sequence were analyzed on a 8 % PAGE gel. The arrow indicates the expected size of the DNA products.

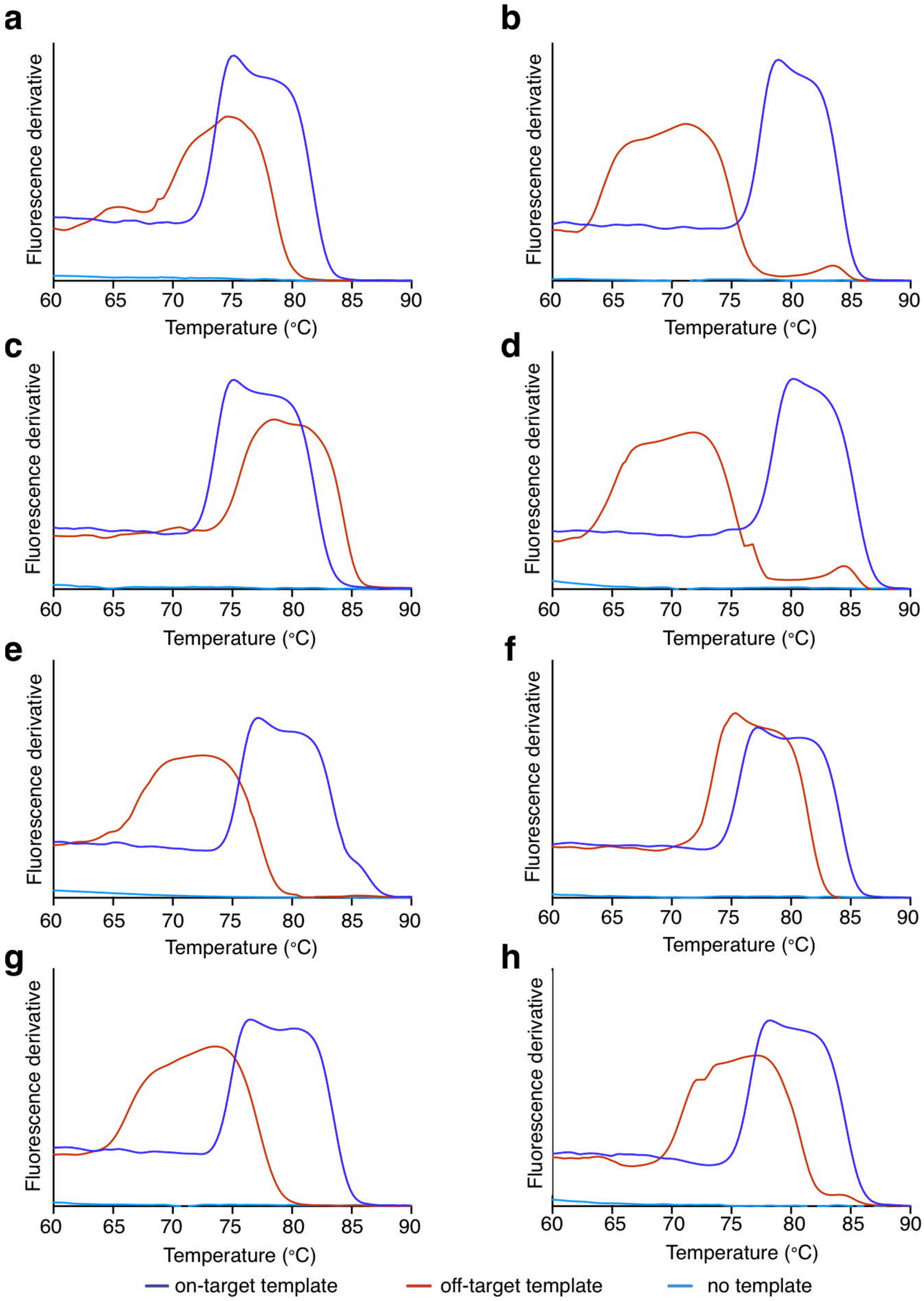

Supplementary Figure 8. Melting curve analysis of SYBR Green qPCR reactions. Melting curves for primer pairs of **(a)** HPV 6, **(b)** HPV 11, **(c)** HPV 16, **(d)** HPV 18, **(e)** HPV 31, **(f)** HPV 33, **(g)** HPV 58, and **(h)** HPV 66 sequences. Each primer set was tested against target template (blue), off-target template from other HPV subtypes (red), or no template control (water, light blue). All measurements were performed in triplicate, and displayed as line plots of the mean fluorescence intensities.

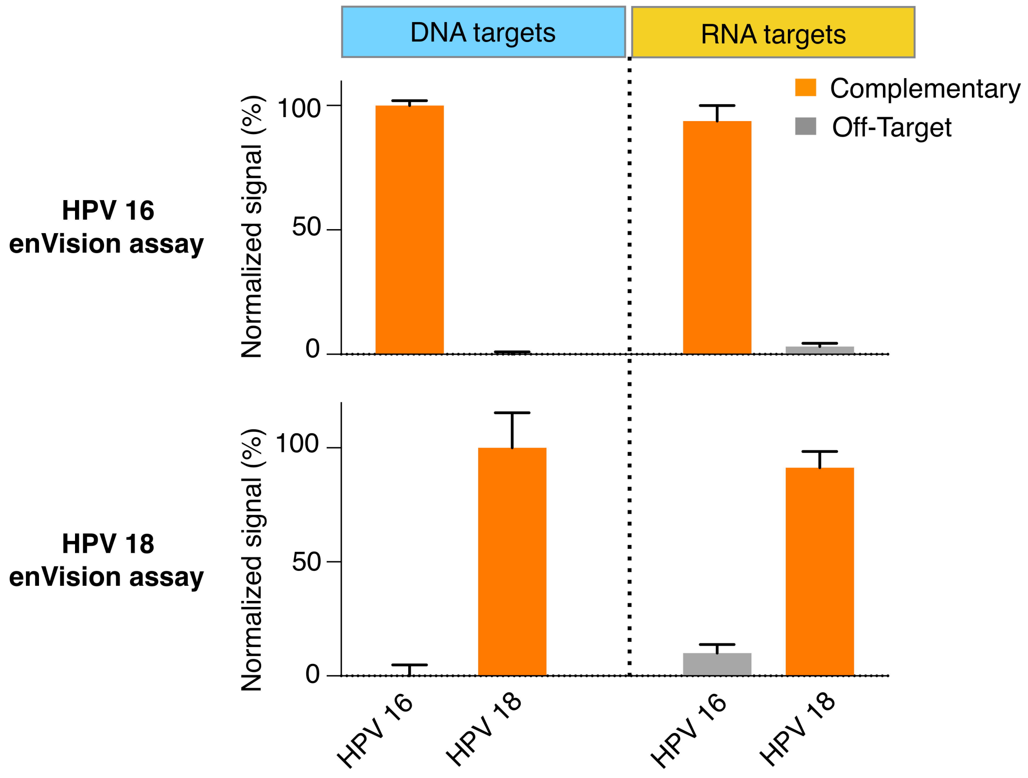

Supplementary Figure 9. Direct RNA detection. We tested the developed enVision assays (i.e., HPV 16 and HPV 18 assays) for direct detection of DNA (left) and RNA (right) of different HPV subtypes. See Methods for details on the preparation of the RNA targets. All RNA targets were used directly without any cDNA conversion. Targets were considered as either complementary or off-target. The enVision assays demonstrated specific and direct detection of RNA targets, with signals comparable to that of DNA targets. All signals were normalized against each assay's positive DNA signal for relative comparison. All measurements were performed in triplicate, and the data are displayed as mean ± s.d.

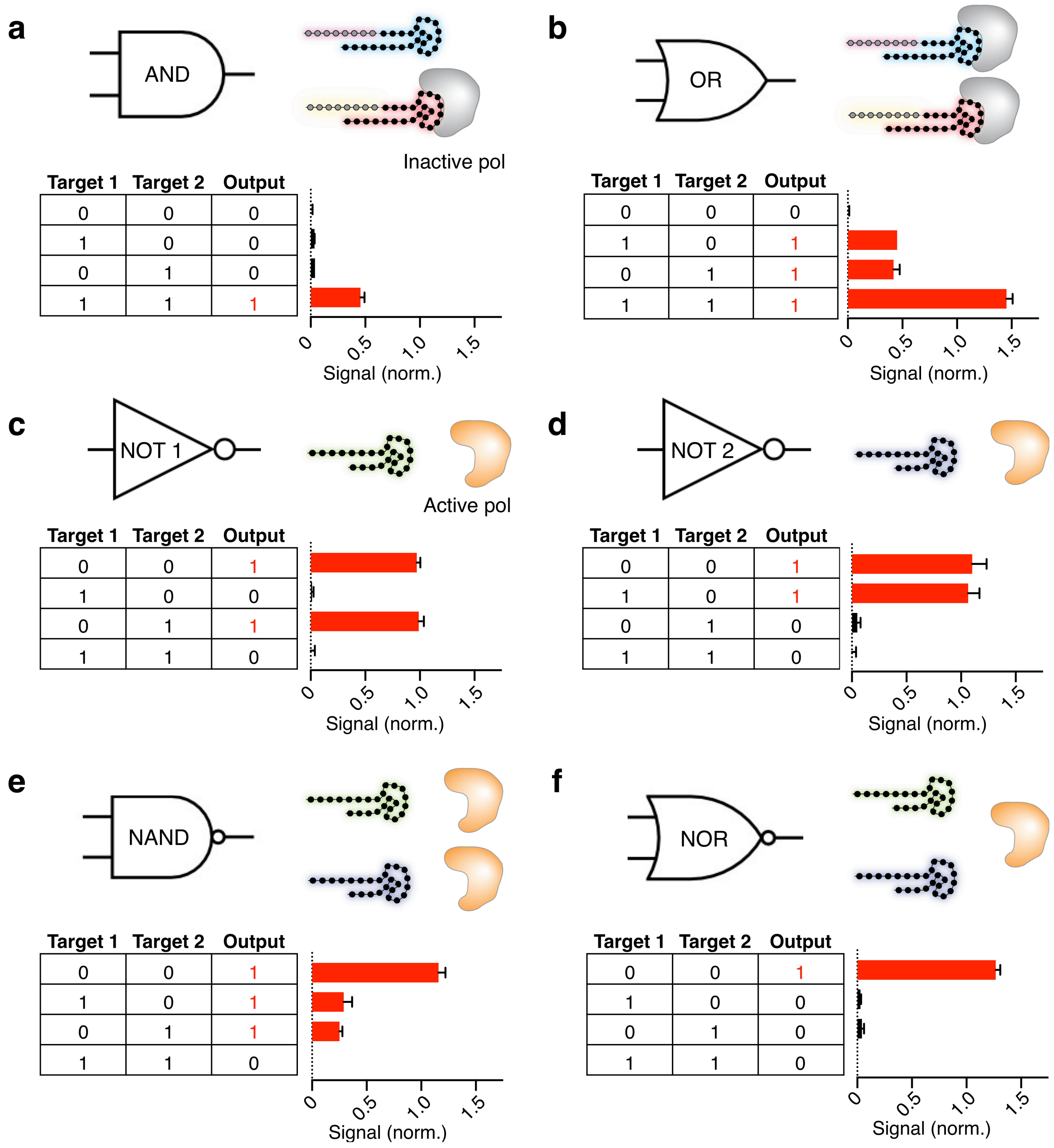

Supplementary Figure 10. enVision logic gates. By varying the combination of recognition nanostructures as well as the ratio of different components (i.e., aptamer, inverter and polymerase) in each nanostructure, we programmed the following logic computations: **(a)** AND gate, **(b)** OR gate, **(c)** NOT gate for HPV 16, **(d)** NOT gate for HPV 18, **(e)** NAND gate, and **(f)** NOR gate. For each gate designed, the components used to establish the configuration are illustrated (top right of each panel). Each gate was tested with different combinations of DNA targets, isolated from HPV 16 (Target 1) and HPV 18 (Target 2). All target combinations and their expected computational outputs are summarized in corresponding truth tables (bottom left of each panel). The observed enVision signals (bottom right of each panel) showed a good agreement with the expected outputs. All signals were normalized to appropriate controls (no-target controls) as previously described. Normalized signals above the detection threshold (i.e., 3x s.d. higher than background signal) were considered as true signals (red bars); otherwise a false signal was called (black bars). All measurements were performed in triplicate, and the data are displayed as mean ± s.d.

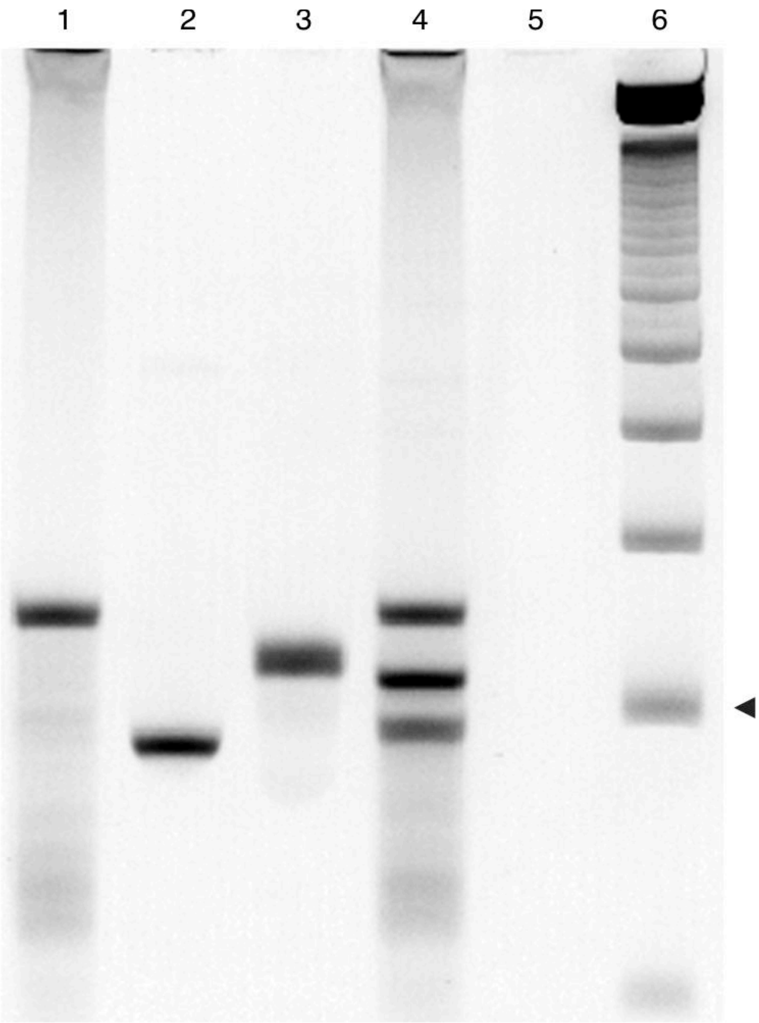

Supplementary Figure 11. Multiplexed amplification of genomic DNA. Full gel electropherogram of amplification products from CaSki genomic DNA. Genomic DNA was amplified in the presence of different HPV 16 locus primers: (1) E1, (2) L1, (3) L2, (4) combined primers of E1, L1 and L2, as well as (5) no primers. Lane 7 was loaded with 15 bp DNA ladder. Arrow indicates the position of the 50 bp band.

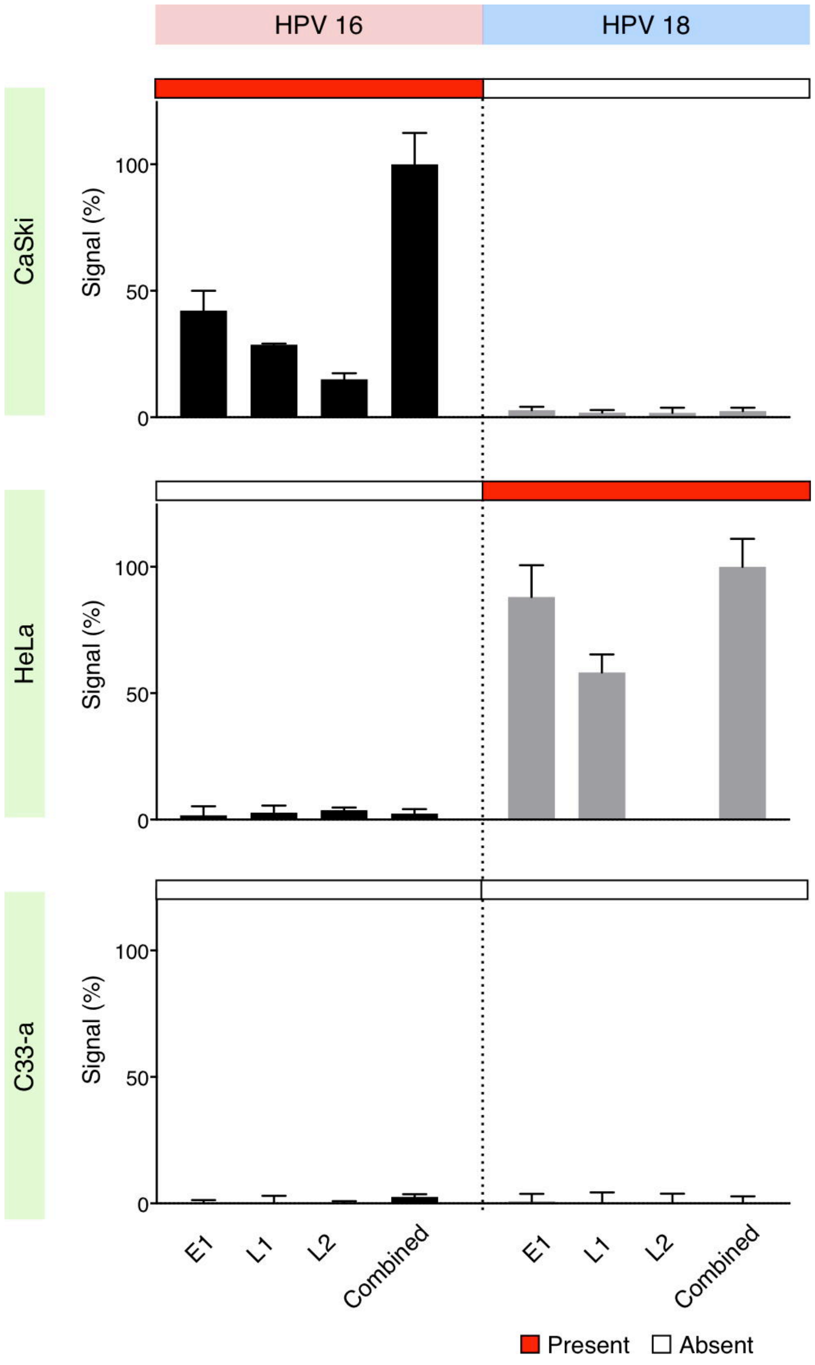

Supplementary Figure 12. Multiplexed enVision detection of genomic DNA. Equal amounts of cellular genomic DNA (top: CaSki, middle: HeLa, bottom: C33-a) were incubated directly with specific recognition nanostructures against different HPV subtypes (left: HPV 16, right: HPV 18). Samples were treated with individual recognition nanostructures (i.e., E1, L1 and L2) or a pool of three structures simultaneously (combined). All signals were normalized as a percentage of the maximal signal observed for each HPV subtype. The multiplexed measurements correlated well with the known HPV infections of the cell lines, as reported by previous literatures (red: present, white: absent). Note that the single locus measurement could miss positive infection (e.g., HeLa, HPV 18 locus L2). All measurements were performed in triplicate, and the data are displayed as mean ± s.d.

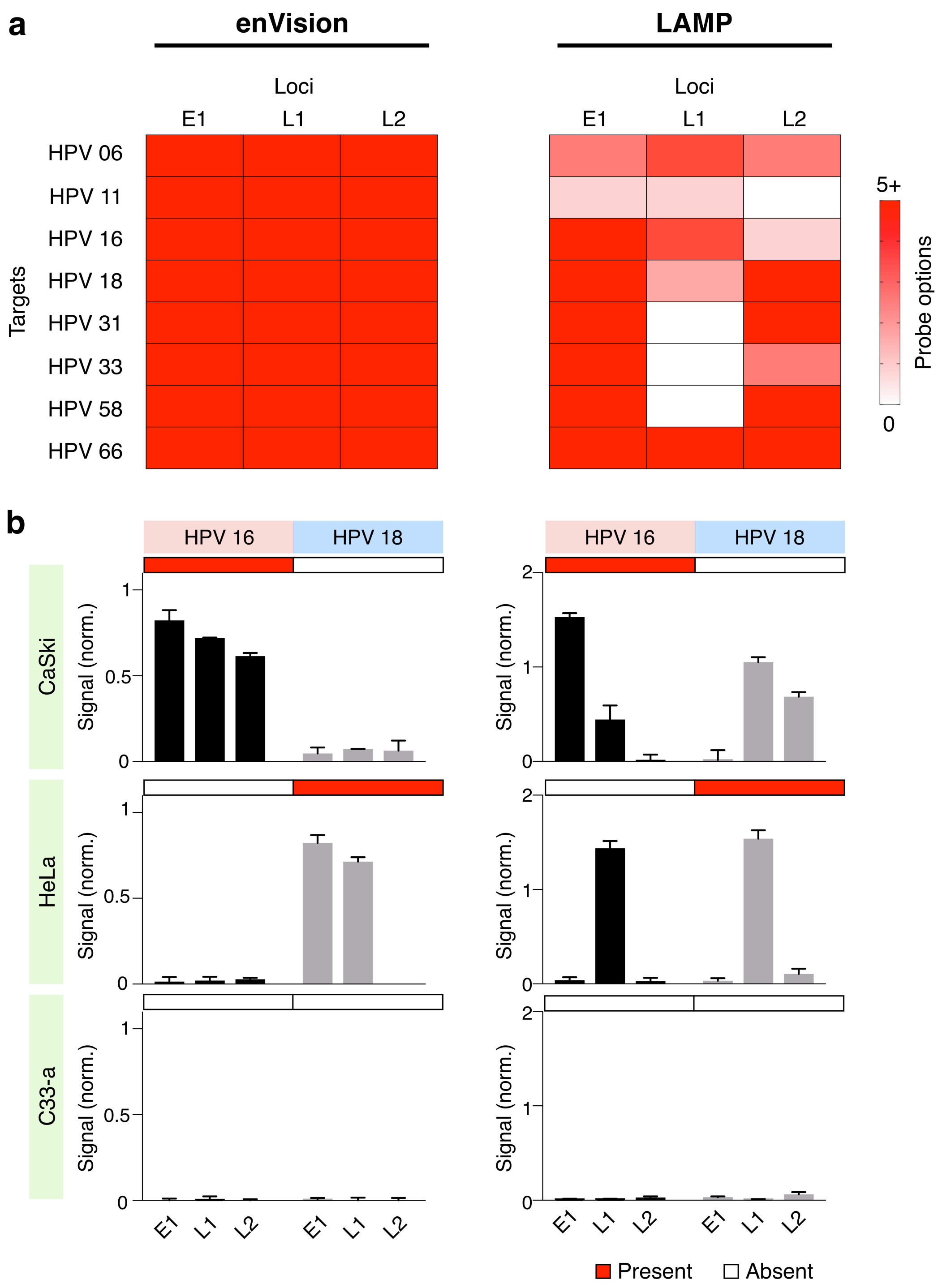

Supplementary Figure 13. Comparison of enVision and LAMP design and performance. (a) Comparison of probe options found in the highly variable regions of the E1, L1 and L2 loci in different HPV subtypes. Probe options were identified for the enVision system (left) as well as LAMP (right). The enVision platform not only generated more probe choices but also provided comprehensive coverage for all regions tested. **(b)** Comparison of enVision performance (left) with that of top-ranked LAMP primer sets (right) for HPV subtyping in cellular genomic DNA. enVision had 83.3% sensitivity (5/6) and 100% specificity (12/12) while LAMP had 50.0% sensitivity (3/6) and 75.0% specificity (9/12). Multi-loci measurements were made across different cell lines of known infections. All signals were normalized to appropriate controls (no-target controls) as previously described. Note that only the enVision technology showed accurate HPV subtyping, as compared to known cellular infection status (red bar: present, white bar: absent). LAMP

demonstrated significant false positives (e.g., CaSki cells, HPV 18; HeLa cells, HPV 16). All measurements were performed in triplicate, and the data are displayed as mean ± s.d.

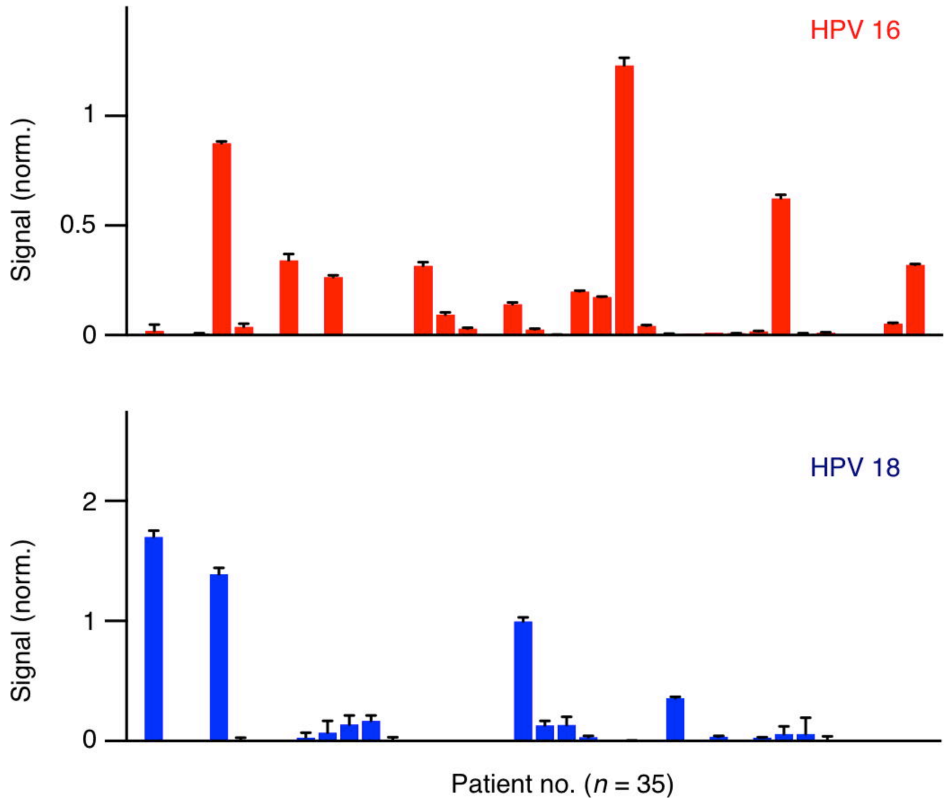

Supplementary Figure 14. Multiplexed enVision detection in clinical samples. High-coverage multi-loci enVision assays (simultaneous detection of E1, L1 and L2) were performed in clinical endocervical brush samples (n = 35 patients) for molecular subtyping of HPV 16 (top panel) and HPV 18 (bottom panel). All measurements were performed in triplicate, and the data are displayed as mean ± s.d.

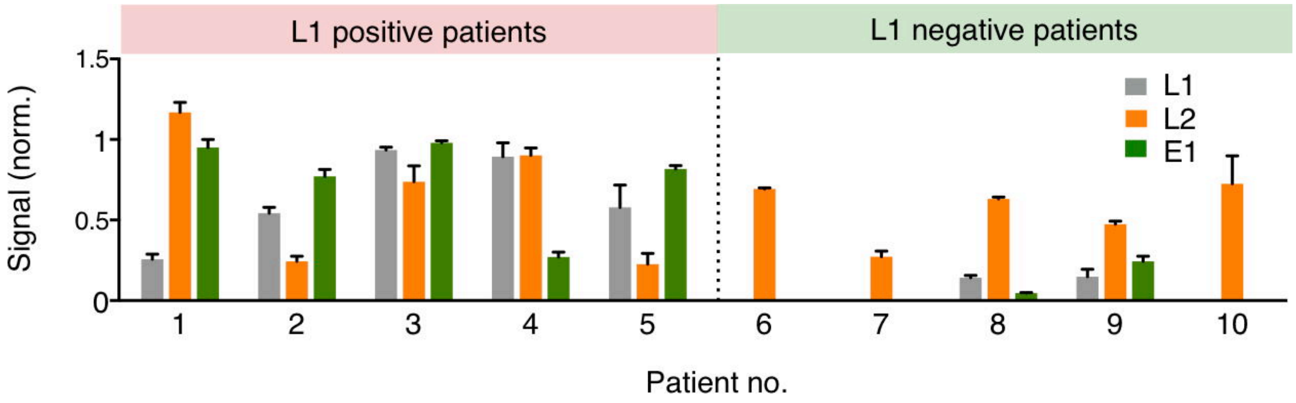

Supplementary Figure 15. Clinical validation with Taqman fluorescence assays. Taqman assays were designed to detect the HPV 16 E1, L1 and L2 loci, respectively. All clinical validation assays were performed with qPCR analysis. Relative quantitation was performed for each sample by normalizing with respective GAPDH expression. Note that the data correlated well to the signals detected with the enVision platform to identify previously undetectable infections (see Fig. 5c). All measurements were performed in triplicate, and the data are displayed as mean ± s.d.

Supplementary Table 1. Oligonucleotides used for activity and sensitivity characterization.

**Nanostructure characterization oligonucleotides**	
Recognition nanostructure characterization	
Aptamer	AAGTATCTGTAATAAAGTCACAATGTACAGTATTG
Inverter	TGACTTTATTACAGATACTTCTACAACCCCGGTACCATCT
Complementary target	AGATGGTACCGGGGTTGTAGAAGTATCTGTAATAAAGTCA
Scrambled target	AGTAGAACGCGATGGTACAGGCACTGCAGGGTCCATGTCA
Signaling nanostructure characterization	
Self-priming template	AGCAGGCAGTTACGGGCTGGTGCGATGAGAGACGCGGAGTGTGGCGGCCGGATAGTAATGACTGCGACCGGTGTACCAGTGGCGTGAGGCAGGTCGTGAGGCGGCGTACGTAGAGCGTTGAGCAGGATGCCAACAGTCGATCAGGACGAGTGCTAACGCATTGTCGATAGCTCAGCTGTCTGAGCTATCGACAATGCGTT
Linear template	AGCAGGCAGTTACGGGCTGGTGCGATGAGAGACGCGGAGTGTGGCGGCCGGATAGTAATGACTGCGACCGGTGTACCAGTGGCGTGAGGCAGGTCGTGAGGCGGCGTACGTAGAGCGTTGAGCAGGATGCCAACAGTCGATCAGGACGAGTGCTAACGCATTGTCGATAGCTCA
Linear template primer	TGAGCTATCGACAATGCGTT
10bp Taqman Probe	/56-FAM/CTGATCGAC/ZEN/TGTTGGCATCC/3IABkFQ/
20bp Taqman Probe	/56-FAM/TTGGCATCC/ZEN/TGCTCAACGCT/3IABkFQ/
30bp Taqman Probe	/56-FAM/TGCTCAACG/ZEN/CTCTACGTACG/3IABkFQ/
50bp Taqman Probe	/56-FAM/GCCGCCTCAC/ZEN/GACCTGCCTC/3IABkFQ/
100bp Taqman Probe	/56-FAM/TATCCGGCC/ZEN/GCCACACTCCG/3IABkFQ/
130bp Taqman Probe	/56-FAM/CGCACCAGC/ZEN/CCGTAACTGCC/3IABkFQ/
5' amine universal signaling nanostructure	/5AmMC12/ GCGGCGTACGTAGAGCGTTGAGCAGGATGCCAACAGTCGATCAGGACGAGTGCTAACGCATTGTCGATAGCTCAGCTGTCTGAGCTATCGACAATGCGTT

Mismatch characterization	
Complementary target overhang 2 mismatch	AGATGGTACCG**C**GGTTGTA**T**AAGTATCTGTAATAAAGTCA
Complementary target overhang 4 mismatch	AGAT**A**GT**G**CCG**C**GGTTGTA**T**AAGTATCTGTAATAAAGTCA
Complementary target overhang 6 mismatch	AGAT**A**GT**G**C**A**G**C**GGTT**A**TA**T**AAGTATCTGTAATAAAGTCA
Complementary target overhang 8 mismatch	A**C**AT**A**GT**G**C**A**G**C**GG**C**T**A**TA**T**AAGTATCTGTAATAAAGTCA
Complementary target overhang 10 mismatch	A**CG**T**A**GT**G**C**A**G**CA**G**C**T**A**TA**T**AAGTATCTGTAATAAAGTCA
Complementary target overhang 12 mismatch	A**CG**T**A**GT**GTA**G**CA**G**C**T**A**T**CT**AAGTATCTGTAATAAAGTCA
Complementary target duplex 2 mismatch	AGATGGTACCGGGGTTGTAGAA**T**TATCTGTAATA**G**AGTCA
Complementary target duplex 4 mismatch	AGATGGTACCGGGGTTGTAGAA**T**TA**G**CTGTA**C**TA**G**AGTCA
Complementary target duplex 6 mismatch	AGATGGTACCGGGGTTGTAG**T**A**T**TA**G**CT**C**TA**C**TA**G**AGTCA
Complementary target duplex 8 mismatch	AGATGGTACCGGGGTTGTAG**T**A**T**T**CG**CT**C**TA**C**TA**G**AGT**A**A
Complementary target duplex 10 mismatch	AGATGGTACCGGGGTTGTAG**T**A**T**T**CG**CT**C**TA**CA**A**G**A**T**T**A**A
Complementary target duplex 12 mismatch	AGATGGTACCGGGGTTGTAG**T**A**T**T**CG**C**AC**TA**CA**A**G**A**T**T**AC**

Note: Mismatched nucleotides are indicated in bold.

**Target amplification demonstration**			
Asymmetric Amplification			
HPV 16 forward primer	ATGGATTATATGATATTTATGC	HPV 16 reverse primer	CTGATAAAGATGTAGAGG
HPV 16 product	CTGATAAAGATGTAGAGGGTACAGATGGTACCGGGGTTGTAGAAGTATCTGTAATAAAGTCATCTGCATAAATATCATATAATCCAT		

NASBA			
HPV 16 NASBA forward primer	AATTCTAATACGACTCACTATAGGGAGAAGGGCAGCCTCACCTACTTCTATTA	HPV 16 NASBA reverse primer	AAAGATGTAGAGGGTACAGA
HPV 16 NASBA product	GCAGCCTCACCTACTTCTATTAATAATGGATTATATGATATTTATGCAGATGACTTTATTACAGATACTTCTACAACCCCGGTACCATCTGTACCCTCTACATCTTT		

Supplementary Table 2. Nanostructures for HPV pan-detection and specific subtyping.

Pan-HPV nanostructure 1			
Aptamer	TTTAAATAATCTGGATATTTCAAT GTACAGTATTG	Inverter	AAATATCCAGATTATTTAAAAATG GCTGCA
Complementary positive target	CATAAGGATCTGCAGCCATTTTTAAATAATCTGGATATTT		
HPV 06 Target	CATATGGGTCTGCAGCCATTTGTAAATAATCTGGATATTT		
HPV 16 Target	CATATGGTTCTGACACCATTTTAATATAATCTGGATATTT		
HPV 18 Target	CATAAGGATCTGCAGACATTTGTAAATAATCAGGATATTT		
HPV 31 Target	CATATGGCTCAGCAACCATTTTAAGATAATCTGGATATTT		
HPV 33 Target	CATATGGCTCAGCAACCATTTTAAGATAATCTGGATATTT		
HPV 58 Target	CATAAGGTTCACTGGCCATTTTTAAATAATCTGGATATTT		
Pan-HPV nanostructure 2			
Aptamer	AAATAATTGTGCCTCAGAGGCA ATGTACAGTATTG	Inverter	CCTCTGAGGCACAATTATTTAATA AACCATATTGGCTACA
Complementary positive target	TGTAGCCAATATGGTTTATTAAATAATTGTGCCTCAGAGG		
HPV 06 Target	TGTAGCCAATATGGCTTATTAAACAATTGTGCCTCAGAGG		
HPV 16 Target	TGTAACCAATAAGGTTTATTGAATATTTGGGCATCAGAGG		
HPV 18 Target	TGTAACCAATATGGTTTATTAAACAACTGGGAGTCAGAGG		
HPV 31 Target	TGCATCCAATATGGTTTATTAAAAATTTGTGCATCTGAAG		
HPV 33 Target	TGTAGCCAATATGGCTTATTAAATAACTGAGATTCGGAAG		
HPV 58 Target	TGTAGCCAATAAGGCTTATTAAATAATTGTGATTCTGAGG		
HPV subtyping			
HPV 06 aptamer	TAATGTCAGGTTCAAAAGATCAA TGTACAGTATTG	HPV 06 inverter	ATCTTTTGAACCTGACATTAACC CTACCCAACACCCTGTT
HPV 06 target	AACAGGGTGTTGGGTAGGGTTAATGTCAGGTTCAAAAGAT		
HPV 11 aptamer	CAGGGATAGGGTCAAATGGTCA ATGTACAGTATTG	HPV 11 inverter	ACCATTTGACCCTATCCCTGACC CTGTCCAACATTCTGTT
HPV 11 target	AACAGAATGTTGGACAGGGTCAGGGATAGGGTCAAATGGT		
HPV 16 aptamer	AAGTATCTGTAATAAAGTCACAA TGTACAGTATTG	HPV 16 inverter	TGACTTTATTACAGATACTTCTAC AACCCCGGTACCATCT
HPV 16 target	AGATGGTACCGGGGTTGTAGAAGTATCTGTAATAAAGTCA		
HPV 18 aptamer	GCACTGCAGGGTCCATGTCAC AATGTACAGTATTG	HPV 18 inverter	TGACATGGACCCTGCAGTGCCT GTACCATCGCGTTCTACT
HPV 18 target	AGTAGAACGCGATGGTACAGGCACTGCAGGGTCCATGTCA		
HPV 31 aptamer	TATCCACAGTAAAATCAGTGCAA TGTACAGTATTG	HPV 31 inverter	CACTGATTTTACTGTGGATACAC CTGCCACACATAATGTT
HPV 31 target	AACATTATGTGTGGCAGGTGTATCCACAGTAAAATCAGTG		
HPV 33 aptamer	TGTGTACATTATCCACATCGCAA TGTACAGTATTG	HPV 33 inverter	CGATGTGGATAATGTACACACCC CAATGCAACACTCATAC
HPV 33 target	GTATGAGTGTTGCATTGGGGTGTGTACATTATCCACATCG		
HPV 58 aptamer	CATGTATAGTATCAGCATCGCAA TGTACAGTATTG	HPV 58 inverter	CGATGCTGATACTATACATGATTT TCAGAGTCCTCTGCAC
HPV 58 target	GTGCAGAGGACTCTGAAAATCATGTATAGTATCAGCATCG		
HPV 66 aptamer	TGGGTGCCTCATCATCAATACA ATGTACAGTATTG	HPV 66 inverter	TATTGATGATGAGGCACCCATTT CATTTCGTCAGTCTGGT
HPV 66 target	ACCAGACTGACGAAATGAAATGGGTGCCTCATCATCAATA		

Supplementary Table 3. SYBR Green qPCR primers and RNA templates.

SYBR Green qPCR for HPV subtyping			
HPV 06 forward primer	GAAGATACATTTGATATTTATGC	HPV 06 reverse primer	ATTAGGTGTGGAAGTTAA
HPV 06 product	GAAGATACATTTGATATTTATGCTGAATCTTTTGAACCTGACATTAACCCTACCCAACACCCT GTTACAAATATATCAGATACATATTTAA		
HPV 11 forward primer	ACACGTTTGATATTTATGC	HPV 11 reverse primer	TATTAGGTGTGGAGGTA
HPV 11 product	ACACGTTTGATATTTATGCTGAACCATTTGACCCTATCCCTGACCCTGTCCAACATTCTGTTA CACAGTCTTATCTTACCTCCACACCTAATA		
HPV 16 forward primer	ATGGATTATATGATATTTATGC	HPV 16 reverse primer	CTGATAAAGATGTAGAGG
HPV 16 product	CTGATAAAGATGTAGAGGGTACAGATGGTACCGGGGTTGTAGAAGTATCTGTAATAAAGTCA TCTGCATAAATATCATATAATCCAT		
HPV 18 forward primer	ACTTGTTTGATATATATGCA	HPV 18 reverse primer	GCGAATATTTAAAAAATGC
HPV 18 product	ACTTGTTTGATATATATGCAGATGACATGGACCCTGCAGTGCCTGTACCATCGCGTTCTACTA CCTCCTTTGCATTTTTTAAATATTCGC		
HPV 31 forward primer	GGCTTATATGACATTTATGC	HPV 31 reverse primer	ACTGTACAGCAGTAGAA
HPV 31 product	GGCTTATATGACATTTATGCAGACACTGATTTTACTGTGGATACACCTGCCACACATAATGTT TCCCCTTCTACTGCTGTACAGT		
HPV 33 forward primer	GTTTGTATGATGTTTATGC	HPV 33 reverse primer	TTGCAAACGTACTGTAT
HPV 33 product	GTTTGTATGATGTTTATGCTGACGATGTGGATAATGTACACACCCCAATGCAACACTCATACA GTACGTTTGCAA		
HPV 58 forward primer	TGGACTTTATGATATTTATGC	HPV 58 reverse primer	GCAAAGGACGTATGT
HPV 58 product	TGGACTTTATGATATTTATGCTGACGATGCTGATACTATACATGATTTTCAGAGTCCTCTGCAC TCACATACGTCCTTTGC		
HPV 66 forward primer	GCCTATATGATATTTATGCA	HPV 66 reverse primer	AGGTAATTGTGCAGAA
HPV 66 product	GCCTATATGATATTTATGCAAATATTGATGATGAGGCACCCATTTCATTTCGTCAGTCTGGTG CTACACCTTCTGCACAATTACCT		

RNA synthesis templates	
HPV 16 L2 template	TCTAATACGACTCACTATAGAGATGGTACCGGGGTTGTAGAAGTATCTGTAATAAAGTCA
HPV 16 L2 template complement	TGACTTTATTACAGATACTTCTACAACCCCGGTACCATCTCTATAGTGAGTCGTATTAGA
HPV 18 L2 template	TCTAATACGACTCACTATAGAGTAGAACGCGATGGTACAGGCACTGCAGGGTCCATGTCA
HPV 18 L2 template complement	TGACATGGACCCTGCAGTGCCTGTACCATCGCGTTCTACTCTATAGTGAGTCGTATTAGA

Supplementary Table 4. Nanostructures for multi-loci HPV detection.

HPV 16			
E1 aptamer	CAACCACCCCCACTTCCACCC AATGTACAGTATTG	E1 inverter	GGTGGAAGTGGGGGTGGTTGCAG TCAGTACAGTAGTGGAA
E1 target	TTCCACTACTGTACTGACTGCAACCACCCCCACTTCCACC		
L1 aptamer	GTTTCTGAAGTAGATATGGCCA ATGTACAGTATTG	L1 inverter	GCCATATCTACTTCAGAAACTACAT ATAAAAATACTAACT
L1 target	AGTTAGTATTTTTATATGTAGTTTCTGAAGTAGATATGGC		
L2 aptamer	AAGTATCTGTAATAAAGTCACA ATGTACAGTATTG	L2 inverter	TGACTTTATTACAGATACTTCTACAA CCCCGGTACCATCT
L2 target	AGATGGTACCGGGGTTGTAGAAGTATCTGTAATAAAGTCA		
HPV 18			
E1 aptamer	TGTGCCCCCGTTGTCTATAGCA ATGTACAGTATTG	E1 inverter	CTATAGACAACGGGGGCACAGAG GGCAACAACAGCAGTGT
E1 target	ACACTGCTGTTGTTGCCCTCTGTGCCCCCGTTGTCTATAG		
L1 aptamer	AGGTACAGGAGACTGTGTAGC AATGTACAGTATTG	L1 inverter	CTACACAGTCTCCTGTACCTGGGC AATATGATGCTACCAA
L1 target	TTGGTAGCATCATATTGCCCAGGTACAGGAGACTGTGTAG		
L2 aptamer	GCACTGCAGGGTCCATGTCAC AATGTACAGTATTG	L2 inverter	TGACATGGACCCTGCAGTGCCTGT ACCATCGCGTTCTACT
L2 target	AGTAGAACGCGATGGTACAGGCACTGCAGGGTCCATGTCA		
Taqman PCR validation			
HPV 16 E1 forward primer	CAACGTGTTGCGATTGGTGT	HPV 16 E1 reverse primer	ACCATTCCCCATGAACATGCTA
HPV 16 E1 Taqman probe	/56-FAM/ACACCCAGT/ZEN/ATAGCTGACAG/3IABkFQ/		
HPV 16 L1 forward primer	CACCTAATGGCTGACCACGA	HPV 16 L1 reverse primer	ACTTGCAGTTGGACATCCCT
HPV 16 L1 Taqman probe	/56-FAM/CACCTACAC/ZEN/AGGCCCAAACC/3IABkFQ/		
HPV 16 L2 forward primer	TTGGAACAGGGTCGGGTACA	HPV 16 L2 reverse primer	GAAGGGCCCACAGGATCTAC
HPV 16 L2 Taqman probe	/56-FAM/TGGGAACAA/ZEN/GGCCTCCCACA/3IABkFQ/		

Supplementary Table 5. Nanostructures for enVision logic gates.

HPV 16 nanostructure sequences	
HPV 16 aptamer	AAGTATCTGTAATAAAGTCACAATGTACAGTATTG
HPV 16 inverter	TGACTTTATTACAGATACTTCTACAACCCCGGTACCATCT
HPV 16 NOT aptamer	TGACTTTATTACAGATACTTCAATGTACAGTATTG
HPV 16 target	AGATGGTACCGGGGTTGTAGAAGTATCTGTAATAAAGTCA
HPV 18 nanostructure sequences	
HPV 18 aptamer	GCACTGCAGGGTCCATGTCACAATGTACAGTATTG
HPV 18 inverter	TGACATGGACCCTGCAGTGCCTGTACCATCGCGTTCTACT
HPV 18 NOT aptamer	TGACATGGACCCTGCAGTGCCAATGTACAGTATTG
HPV 18 target	AGTAGAACGCGATGGTACAGGCACTGCAGGGTCCATGTCA

**Logic gate components (relative ratios)**				
AND Gate				
HPV 16 Aptamer	HPV 16 Inverter	HPV 18 Aptamer	HPV 18 Inverter	Taq Polymerase
1	1	1	1	1
OR Gate				
HPV 16 Aptamer	HPV 16 Inverter	HPV 18 Aptamer	HPV 18 Inverter	Taq Polymerase
1	1	1	1	2
HPV 16 NOT Gate				
HPV 16 NOT Aptamer				Taq Polymerase
1				1
HPV 18 NOT Gate				
		HPV 18 NOT Aptamer		Taq Polymerase
		1		1
NAND Gate				
HPV 16 NOT Aptamer		HPV 18 NOT Aptamer		Taq Polymerase
1		1		2
NOR Gate				
HPV 16 NOT Aptamer		HPV 18 NOT Aptamer		Taq Polymerase
1		1		1

Supplementary Table 6. Top-ranked LAMP primers for different HPV loci.

HPV 16 E1 LAMP primer set ***ΔG:-2.32 kcal/mol***			
HPV 16 E1 LAMP Forward Internal Primer	TGGCGCCCTTCTACCTGTAAACA GCGGGTATGGCAATA	HPV 16 E1 LAMP Back Internal Primer	CACCATGTAGTCAGTATAGTGG TGTTTCACTAACACCCTCTCC
HPV 16 E1 LAMP Forward Primer 3	AGAGCTGCAAAAAGGAGA	HPV 16 E1 LAMP Back Primer 3	GTGTTTGGCATATAGTGTGTC
HPV 16 L1 LAMP primer set ***ΔG:-2.33 kcal/mol***			
HPV 16 L1 LAMP Forward Internal Primer	TGGCAGCACATAATGACATATTTG TGGTAACCAACTATTTGTTACTGT	HPV 16 L1 LAMP Back Internal Primer	AACTTTAAGGAGTACCTACGAC ATGAGTTAAGGTTATTTTGCACA GT
HPV 16 L1 LAMP Forward Primer 3	CCACAATAATGGCATTTGTTG	HPV 16 L1 LAMP Back Primer 3	ATGTATGTATGTCATAACGTCTG
HPV 16 L2 LAMP primer set ***ΔG:-2.27 kcal/mol***			
HPV 16 L2 LAMP Forward Internal Primer	CCGGGGTTGTAGAAGTATCTGTA ATCTCACCTACTTCTATTAATAATG GA	HPV 16 L2 LAMP Back Internal Primer	TACCATCTGTACCCTCTACATCT TTGGAATATTGTATGCACCACCA
HPV 16 L2 LAMP Forward Primer 3	CATATACTACCACTTCACATGC	HPV 16 L2 LAMP Back Primer 3	AATGGGTATATCAGGACCTG
HPV 18 E1 LAMP primer set ***ΔG:-2.03 kcal/mol***			
HPV 18 E1 LAMP Forward Internal Primer	TGAATCTGTGTTGCTTCCACTTCG CGGCTGTTTACAATATCAGA	HPV 18 E1 LAMP Back Internal Primer	AACATGGCGGCAATGTATGTAG TGTCTACACTGCTGTTGTTG
HPV 18 E1 LAMP Forward Primer 3	TAGTGGGCAGAAAAAGGC	HPV 18 E1 LAMP Back Primer 3	ATTGCTATTGTCACTTGTACC
HPV 18 L1 LAMP primer set ***ΔG:-2.33 kcal/mol***			
HPV 18 L1 LAMP Forward Internal Primer	AATCATATTCCTCAACATGTCTGC TCTCCTGTACCTGGGCAAT	HPV 18 L1 LAMP Back Internal Primer	ACTTTAACTGCAGATGTTATGTC CTACCAAAGTTCCAATCCTCTA
HPV 18 L1 LAMP Forward Primer 3	CAATATGTGCTTCTACACAGT	HPV 18 L1 LAMP Back Primer 3	TCCACCAAACTAGTAGTTGG
HPV 18 L2 LAMP primer set ***ΔG:-2.11 kcal/mol***			
HPV 18 L2 LAMP Forward Internal Primer	AAGGAGGTAGTAGAACGCGATGC TTGTTTGATATATATGCAGATGAC	HPV 18 L2 LAMP Back Internal Primer	TGCATTTTTTAAATATTCGCCCA CTGGAGGTTAAAGGGACCGT
HPV 18 L2 LAMP Forward Primer 3	CTTTAGTATCTGCCACGGA	HPV 18 L2 LAMP Back Primer 3	TACAGGCACATCCCAAGA

Top-ranked LAMP primers were determined from their ΔG, the change in free energy during LAMP reaction.

Supplementary Table 7. Comparison of detection technologies.

	enVision	PCR (SYBR, Taqman, e.g., Cobas HPV)	Isothermal amplification (LAMP)	Hybridization
**Detection limit**	High femtomole *(without target preamplification)*, attomole *(with target preamplification)*	High attomole[1]	High attomole[2]	Low nanomole[3]
**Sensitivity (95% CI)**	High 94.7%–100%	Moderate to high 63.1%–100%	Low 43.5%–76.9%	High 85.2%–98.1%[4]
**Specificity (95% CI)**	High 93.6%–100%	Moderate to high 83.0%–98.1%	Moderate 52.3%–93.5%	Moderate to high 89.9%–91.4%[4]
**Target options**	DNA and RNA	DNA	DNA	DNA and RNA
**Sequence design stringency**	Low *single 20–40 base sequence*	Moderate *pair of 18–25 base sequences*	High *sets of four or six 25–35 base sequences*	Low *single 20–40 base sequence*
**Versatility**	High	Moderate	Low	Moderate
**Robustness**	High	Moderate to high	Low	Moderate
**Time taken**	as little as 30 min	~ 2 h	~ 1 h	>2 h, multiple washes
**Temperature requirement**	Isothermal	Thermal cycling	Isothermal	Isothermal
**Equipment requirement**	Minimal (smartphone)	High (thermocycler and fluorometer)	Moderate (fluorometer)	Moderate (fluorometer or colorimeter)
**Ease of use**	Minimal training	Trained personnel	Trained personnel	Trained personnel
**Cost/reaction**	<$1	$1-3	<$1	$1-2

CI, confidence interval.

SUPPLEMENTARY REFERENCES

1. Rao, A. et al. Development and Characterization of the cobas Human Papillomavirus Test. *J Clin Microbiol* **51**, 1478-1484 (2013).
2. Lucchi, N. W. et al. Evaluation of the Illumigene Malaria LAMP: A Robust Molecular Diagnostic Tool for Malaria Parasites. *Sci Rep* **6**, 36808 (2016).
3. Lee, J. S., Song, J. J., Deaton, R. & Kim, J.-W. Assessing the Detection Capacity of Microarrays as Bio/Nanosensing Platforms. *Biomed Res Int* **2013**, 8 (2013).
4. Kang, L. N. et al. Optimal positive cutoff points for careHPV testing of clinician- and self-collected specimens in primary cervical cancer screening: an analysis from rural China. *J Clin Microbiol* **52**, 1954-1961 (2014).
